# A versatile *Bacillus* esterase reveals a carboxylesterase blueprint for PET intermediate degradation

**DOI:** 10.1007/s00253-026-14056-w

**Published:** 2026-10-09

**Authors:** Hadjira Bounabi, Bilal Yahiaoui, Meriem Gasmi, Meroua Safa Mechouche, Nico Bäse, Marno Gurschke, Rebecka Molitor, Pablo Pérez-García, Wolfgang R. Streit

**Affiliations:** 1 Department of Microbiology and Biotechnology, University of Hamburg, Ohnhorststrasse 18, 22609 Hamburg, Germany; 2 Biotechnology Laboratory, Higher National School of Biotechnology Taoufik Khaznadar, Nouveau Pôle Universitaire Ali Mendjeli, 25100 Constantine, Algeria; 3 Laboratory of Applied Microbiology, Department of Microbiology, Faculty of Natural and Life Sciences, Ferhat Abbas University, 19000 Setif, Algeria; 4 Institute of Molecular Enzyme Technology, Heinrich-Heine-University Düsseldorf, Forschungszentrum Jülich, Jülich, Germany

**Keywords:** PETase, BHETase, MHETase, Terephtalate, Enzyme kinetics, Plastic biodegradation

## Abstract

**Abstract:**

The biodegradation of polyethylene terephthalate (PET), and its derived intermediate breakdown products, represents a core focus of multidisciplinary research. While *Ideonella sakaiensis* PETase (IsPETase) and leaf-compost cutinase (LCC) efficiently depolymerize PET, many other PET-active hydrolases display stronger activity toward soluble PET intermediates such as bis- and mono(2-hydroxyethyl) terephthalate (BHET and MHET). Here, we describe a versatile carboxylesterase derived from *Bacillus halotolerans* (BhEstB). BhEstB is structurally related to BsEstB from *B. subtilis* but divergent from canonical PETases and most characterized MHETases, including IsMHETase, while clustering more closely with carboxylesterases such as TfCa. BhEstB displayed broad esterase activity toward *p*NP-esters, with maximum activity at pH 8 and 20–30 °C. Although it showed modest overall activity on the bulk polymer, BhEstB significantly outperformed the engineered variant LCC^ICCG^ and the recently published archaeal feruloyl esterase PET46 in turning over BHET and MHET. BhEstB exhibits remarkably high activity toward BHET and MHET, achieving complete conversion of 50 mM BHET within 1 h at 30 °C (kcat = 15.57 ± 1.25 s⁻^1^, Km = 6.54 ± 1.01 mM, corresponding to a catalytic efficiency of 2.38 × 10^3^ M⁻^1^ s⁻^1^) and subsequent efficient turnover of 39.8 mM MHET (77% (± 0.7%)) into terephthalic acid (TPA) within 24 h. These results identify BhEstB as one of the most efficient BHET-hydrolyzing enzymes characterized to date. Collectively, these findings establish BhEstB as a versatile BHET/MHET active *Bacillus* carboxylesterase with modest activity toward commercial semi-crystalline PET powder, contributing to the expanding diversity of PET-intermediate-degrading enzymes.

**Keypoints:**

• *BhEstB exhibits structural divergence from canonical PET esterases.*

• *BhEstB efficiently degrades significant amounts of BHET and MHET at 30 °C.*

• *BhEstB demonstrates moderate activity on PET powder at elevated temperatures.*

**Graphical Abstract:**

**Figure:**
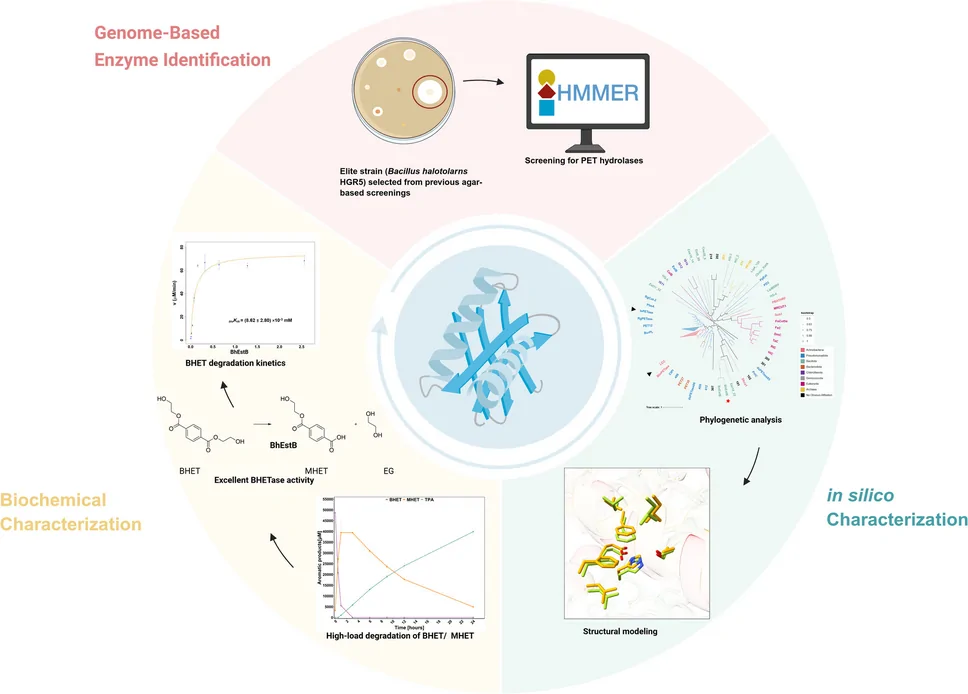

**Supplementary Information:**

The online version contains supplementary material available at https://doi.org/10.1007/s00253-026-14056-w.

## Introduction

Polyethylene terephthalate (PET) is an aromatic polyester derived from petrochemical feedstocks that is widely utilized across the commodity plastics sector due to its lightweight nature, exceptional durability, and water resistance. Consequently, PET hydrolases (commonly termed “PETases”) have garnered significant attention in both academic research and industrial biotechnology for their potential application in enzymatic depolymerization and closed-loop upcycling. Multiple studies have established that PET can be effectively hydrolyzed at temperatures surpassing its glass transition temperature (${T}_{g}$), which spans approximately 70–80 °C in air and decreases by roughly 10 °C in aqueous environments. At these elevated temperatures, the amorphous regions of the polymer chains exhibit increased chain mobility and flexibility, rendering the identification and engineering of robust, thermophilic PET hydrolases a primary objective in environmental biotechnology (Wei et al. 2022; Tournier et al. 2023; Pérez-García et al. 2025) and references therein.

To date, more than 300 PET-degrading enzymes have been discovery-screened or biochemically characterized (Buchholz et al. 2022; see the Plastics-Active Enzymes Database [PAZy], www.pazy.org). Among these, the leaf-compost cutinase (LCC) represents one of the most thoroughly evaluated model systems and is widely considered one of the most active wild-type scaffolds identified to date (Sulaiman et al. 2012). Another paradigm is the PETase from *Ideonella sakaiensis* (*Is*PETase), a Gram-negative bacterium originally reported to utilize PET as a primary carbon and energy source (Yoshida et al. 2016). Recently, tailored variants of both *Is*PETase and LCC developed via rational design and directed evolution have shown significantly accelerated depolymerization rates, underscoring the vital role of protein engineering in advancing scale-up recycling workflows. Notably, the engineered variant ${LCC}^{ICCG}$ achieved industrial-scale PET depolymerization, enabling a true closed-loop circular economy via efficient monomer recovery and subsequent repolymerization (Tournier et al. 2020; Lu et al. 2022; Bell et al. 2022).

Although these advanced, engineered biocatalysts efficiently depolymerize bulk PET under industrially viable conditions, their kinetic efficiency is heavily bottlenecked by the accumulation of intermediate breakdown products. Specifically, mono-(2-hydroxyethyl) terephthalate (MHET) acts as a potent competitive inhibitor to primary endo-type PET hydrolases, necessitating complex multi-enzyme cocktails or two-step bioreaction setups (Liu et al. 2026; Lu et al. 2026). While specialized MHETases successfully alleviate this inhibition, their specialized lid architectures confer pronounced substrate specificity toward monomeric intermediates, while providing little or no activity toward polymeric PET (Palm et al. 2019). Current research indicates that, with the exception of the mesophilic IsMHETase, most characterized MHETases exhibit low affinity and catalytic efficiency for MHET. Moreover, IsMHETase engineering remains challenging due to its complex architecture, expression and solubility constraints, limited screening methods9, and incomplete mechanistic understanding, which together impede predictive engineering and industrial optimization. Hence, enhancing MHETase activity is essential for efficient industrial PET degradation (Liu et al. 2026).

The *Ideonella sakaiensis* MHETase (*Is*MHETase, PDB-ID 6QZ1) is one of the most thoroughly characterized MHETases, alongside the bathyarchaeal enzyme PET46, the carboxylesterase TfCa (PDB-ID: 7W1K), the bacterial thermostable esterase MHT077, and many others (Lu et al. 2026). *Is*MHETase belongs to the tannase family and exhibits a temperature optimum of 30–50 °C (Sagong et al. 2020). Conversely, PET46 (PDB-ID: 8B4U) is a highly thermostable feruloyl esterase derived from *Candidatus* Bathyarchaeota (Perez-Garcia et al. 2023), while MHT077 (PDB-ID: 9PIC) belongs to the dienelactone hydrolase superfamily and originates from the bacterial phylum Armatimonadota (Murphy et al. 2026).

Interestingly, while LCC, *Is*PETase, and most other PET-active hydrolases can process MHET, they strongly prefer the polymeric substrate. In contrast, *Is*MHETase, MHT077, and PET46 exhibit moderate-to-low exopolymer activities but are more efficient in the turnover of monomeric MHET and dimeric BHET (Murphy et al. 2026; Pérez-García et al., 2023; Sagong et al. 2020). This functional divergence may be linked to their distinct evolutionary origins and the presence of a lid domain, which creates steric hindrance that limits the access of bulk polymer chains to the catalytic active site. Within this framework, we investigated whether we could identify highly active PET hydrolases that simultaneously possess significant MHETase or BHETase activity.

The phylum *Bacillota* contains 18 putative PET esterases indexed in the Plastics-Active Enzymes Database (PAZy), all of which share low amino acid sequence similarity with characterized clusters from Pseudomonadota and Actinomycetota. Furthermore, these *Bacillota*-derived candidates generally exhibit low baseline activity on bulk PET compared to LCC (Ribitsch et al. 2011; Li et al. 2023; Vidal et al. 2024). Among them, two enzymes belong to the genus *Bacillus*: an esterase (BsEst) from *Bacillus subtilis* and a lipase (LipMRD9) from *Bacillus safensis*. Notably, BsEst has shown remarkably high activity toward bis(2-hydroxyethyl) terephthalate (BHET), making it an intriguing scaffold for further optimization. BsEst in its engineered form (ΔBsEst) had a catalytic efficiency (kcat/KM) that was 3.5-fold improved and ΔBsEst had a 100% BHET conversion in 3 h, which is a twofold improvement over the wild type and when 5 mM substrate was supplied (at 30 °C) (Li et al. 2023).

Based on these earlier studies, we investigated whether other *Bacillus*-derived enzymes share similar traits, and whether enzymes from this genus generally function as multi-substrate “all-rounders” possessing concurrent activities against PET, BHET, and MHET. To explore this, we mined the genome of a recently isolated, halotolerant *Bacillus* strain (HGR5) and identified an esterase displaying activity toward both the polymer and its intermediate breakdown products.

Here, we report the biochemical characterization of the versatile, *Bacillus*-derived esterase, designated BhEstB (GenBank-ID: PZ568891). Structurally, BhEstB is a highly conserved *para*-nitrobenzyl esterase that carries a lid domain. The purified recombinant enzyme exhibits excellent catalytic efficiency against BHET, fully converting 50 mM BHET within 1 h and substantially hydrolyzing nearly 40 mM MHET at 30 °C in 24 h. Furthermore, BhEstB hydrolyzes accessible ester bonds in semi-crystalline PET powder at elevated temperatures (producing up to 689.5 µM soluble products). Together, these findings provide key biochemical insights into PET intermediate hydrolysis by *Bacillota* carboxylesterases.

## Material and methods

### Bioinformatic analysis

The protein database of *Bacillus halotolerans* HGR5 used in this study was constructed from its genome, which is available in GenBank under the accession number JASXRX000000000 (Yahiaoui et al. 2023). Sequences of PET-degrading enzymes were retrieved from the PAZy database and aligned using MUSCLE implemented in MEGA11. HMMER v3.4 tools, hmmbuild and hmmsearch, were then applied as previously described to construct an HMM from the alignment and to search the strain’s protein database (Chow et al. 2023). Phylogenetic analysis was constructed in MEGA11 using the maximum likelihood method with 500 bootstrap replicates. The generated tree was exported in Newick format and subsequently visualized and edited using iTOL platform (Letunic and Bork 2024). The sequence of BhEstB is available in GenBank under accession number PZ568891.

CD-Search (Conserved Domain Search; (Marchler-Bauer et al. 2015)) and SignalP (Nielsen 2025) were used to identify conserved domains and signal peptides in BhEstB. AlphaFold2 (Jumper et al. 2021) was used to determine the 3D structure of BhEstB. Protein+ (Ehrt et al. 2025) and ConSurf (Ben Chorin et al. 2020) servers were used to predict substrate binding pocket and the conservation level of amino acids, respectively. Moreover, BHET, MHET, and 3PET were docked into the catalytic site of BhEstB via Attracting Cavities 2.0 docking engine (Bugnon et al. 2024). Structural visualization and alignments were all performed using USFC ChimeraX v.1.9 (Meng et al. 2023). Bulk structural comparison was performed on the Dali server (Holm et al. 2023). The resulting similarity matrix was used to create a heatmap in R v.4.4.1 using the pheatmap package v1.0.12.

### DNA sequencing, gene cloning, and expression

The genomic DNA of *B. halotolerans* HGR5 was isolated using the NucleoSpin Microbial DNA kit (MACHEREY–NAGEL, Düren, Germany) following the manufacturer’s instructions. The sequence of the gene coding for BhEstB was amplified via PCR and cloned in pET21a(+) (Novogene, Cambridge, UK) using NheI and HindIII restriction sites and transformed in *Escherichia coli* BL21(DE3) (Novagen/Merck, Darmstadt, Germany) for protein production. The primers used were synthesized by Eurofins Genomics (Ebersberg, Germany) and are listed in supplementary note 1 along with the DNA and the protein sequences. Sequencing of the plasmid was conducted by Microsynth Seqlab GmbH (Göttingen, Germany).

### Protein expression and purification

*E. coli* BL21(DE3) cells carrying pET21a(+) constructs with either of the genes coding for BhEstB, LCC^WT^, LCC^ICCG^, or PET46; or pMAL-p4X construct with genes coding for IsPETase or IsMHETase were used to express these enzymes in autoinduction medium (Studier 2005) containing 100 µg/mL ampicillin at 37 °C. At OD_600_ 0.7, cultures were transferred to 22 °C for overnight protein production. The cells were centrifuged (9722*g*; Sorvall RC 6 +, ThermoFisher Scientific, Waltham, MA, USA) and washed twice before being lysed three times using French Press Cell Press (American Instrument Company) at 1250 psi. Following a second centrifugation, the enzymes were purified from the supernatant with Ni-NTA Agarose (Macherey-Nagel, Düren, Germany) or Amylose (New England Biolabs (NEB), Ipswich, MA, USA) columns according to the manuals. The purified enzymes were concentrated and dialyzed against 100 µM potassium phosphate buffer pH 8.

### Biochemical characterization and PET degradation assays

An initial biochemical characterization of BhEstB was conducted using *para*-nitrophenyl (*p*NP) esters across a range of chain lengths (C4–C18), temperatures (4–70 °C), and pH (4–11), enabling determination of substrate preference and optimal temperature and pH. Experiments were carried out as reported in other works (Zhang et al. 2022).

To evaluate BHET degradation, 0.06 µM of enzyme was incubated with 300 µM substrate at either 30, 40, 50, 60, or 70 °C for 1 h. Enzyme kinetics toward BHET were determined by measuring the initial reaction rates at substrate concentrations ranging from 0.1 to 15 mM and enzyme concentrations from 0.005 to 2.56 µM for conventional and inverse kinetics, respectively. The initial velocities were measured and plotted against BHET concentrations. Furthermore, the degradation of a high concentration of BHET (50 mM) by various enzymes was investigated over a 24-h period, using an enzyme concentration of 0.09 mg/mL. Samples were collected at multiple time points to monitor the progression of substrate degradation. Furthermore, the degradation 50 µM ethylene terephthalate linear trimer (3PET, Toronto Research Chemicals, Ontario, Canada) was also assessed using 3 µM of the same enzymes over 24 h (Perez-Garcia et al. 2023).

Moreover, 3 µM BhEstB, IsPETase, LCC^WT^, and LCC^ICCG^ were incubated with 7 mg amorphous PET foil platelet (product no. ES301445; a = 5 mm^2^, 33.6 µmol or 168 mM TPA eq.; GoodFellow GmbH, Hamburg, Germany) and 7 mg semi-crystalline PET powder (product no. ES306000; > 40% crystallinity, d≈300 µm; 9.6 µmol or 168 mM TPA eq.; GoodFellow GmbH, Hamburg, Germany). The reaction took place under agitation (450 rpm) at 30, 40, 50, 60, or 70 °C for 24 h.

On the other hand, to evaluate MHET degradation by BhEstB, IsMHETase, PET46, and LCC^ICCG^, PET powder degradation reaction by 3 µM LCC^ICCG^ was interrupted after 4 h, by vortexing and centrifugation (15,000*g*, Centrifuge 5417R, Eppendorf, Hamburg, Germany). The supernatant of multiple tubes was collected and served as the substrate sample. Enzyme stocks of 50 µM were diluted 1:100 in the supernatant to a final concentration of 0.5 µM and incubated at different temperatures for the remaining time up to 24 h. A control, diluted in the potassium phosphate buffer, was prepared and run in parallel under the same conditions. Samples were taken at various time points to monitor the degradation.

All assays described were carried out in triplicate, with enzyme-free controls performed in parallel for each. All data were corrected against the controls. Where appropriate, Welch’s two-sample *t*-test was used to assess statistical significance. Unless otherwise specified, all experiments were conducted in 100 µM potassium phosphate buffer (PPB) pH 8 at a final volume of 200 µL under agitation (450 rpm).

### Optimization of the biodegradation

Enzymatic degradation of semi-crystalline PET powder by BhEstB was optimized using Central Composite Design (CCD); the selected factors were temperature (X1), pH (X2), and enzyme concentration (X3; Table 1). Fourteen combinations were possible, each representing a combination of the different levels of the selected factors. Additionally, 4 center point experiments were included, all of which were performed in triplicate. Hence, 54 experiments were conducted in total, according to the protocol described above. Table 1 shows the levels of the studied variables; all the other details are provided in the supplementary data. The MINITAB software v17 was used to generate the matrix, analyze the outcomes, validate the model, and develop a second-order polynomial equation using response surface methodology (RSM).

**Table 1:** Real and coded values of independent variables used for calculated optimization of PET degradation

Level	− 2	− 1	0	+ 1	+ 2
Temperature (°C)	55	60	65	70	75
pH	7	7.5	8	8.5	9
[BhEstB] (µM)	1	1.5	2	2.5	3

The nonlinear optimization using an Artificial Neural Network coupled with a Genetic Algorithm (GA) was performed in MATLAB R2024 (MathWorks, Inc., Massachusetts, USA) as described by Mechouche et al. (2024). Briefly, the experimental matrix developed from RSM was employed in constructing input and output vectors for the neural network model training, and the data generated by the ANN model served as the initial population for the GA. Validation experiments were performed at the ANN/GA-predicted optimum conditions to confirm the model prediction. The absolute percentage error (%) between the predicted and experimental results was calculated to check the model’s accuracy (Eq. 1).

1 $$absolute\; error\; \left(\%\right)= \frac{\left|{Y}_{i}-\widehat{{Y}_{i}}\right|}{Yi} \times 100$$

### Quantification of degradation products via ultra-high-performance liquid chromatography (UHPLC)

BHET, 3PET and PET degradation were evaluated by UHPLC analysis to quantify various aromatic products including BHET, MHET, and TPA via UV detection. Initially, the samples underwent homogenization and centrifugation to ensure they were properly prepared for measurement. A 96-well microtiter plate was used to prepare samples; 12.5 µL reaction supernatant was added to 50 µL acetonitrile with 1% v/v trifluoroacetic acid (TFA) followed by centrifugation at 2,204 g for 3 min (Centrifuge 5804R, Eppendorf, Hamburg, Germany). Afterward, 50 µL of centrifugation supernatant was transferred into 150 µL MilliQ H_2_O. Plates were covered using ZoneFree™ sealing film (Excel Scientific, Victorville, CA, USA) and used for analysis via RP-UHPLC (UltiMate™ 3000 UHPLC system, Thermo Scientific, Waltham, MA, USA) with a Triart C18 column (YMC Europe GmbH, Dinslaken, Germany) or stored at −20 °C until analysis. A mobile phase consisting of 20:80 (v/v) acetonitrile and water (acidified with 0.1% v/v TFA) was used at a flowrate of 0.4 mL/min. Standards of TPA, MHET, and BHET were also prepared and measured to determine the respective elution times and construct a calibration curve.

### Measurement of protein thermal stability

Purified enzyme prepared at 1 mg/mL was filled into NanoTemper capillary tubes, which were then placed into a Prometheus nanoDSF (NanoTemper Technologies GmbH, Munich, Germany). A melting scan with a range from 20 to 95 °C with a heating rate of 1 °C/min was applied and the results were analyzed with the NanoTemper software.

## Results

### Hidden-Markov model (HMM) search reveals BhEstB as a candidate PET hydrolase

To identify potential PETases with high MHETase and BHETase activities, we set out to reevaluate a previously identified collection of 72 novel bacterial strains from terrestrial hot springs in Algeria. These strains had been identified because of their abilities to degrade diverse natural polymers (Yahiaoui et al. 2023). Among these, we identified one bacterium that displayed weak polyesterase activity. The selected strain was named *Bacillus halotolerans* HGR5 and its genome sequence was reported (Yahiaoui et al. 2023). Further experimental work revealed that this bacterium was promising with respect to plastic polymer degradation and was therefore selected for an in-depth characterization. An HMM search identified 19 potential PETase open reading frames (ORFs) with a score from 186 down to 14. The hit with the highest score signaled a potential PET activity, and it was named BhEstB (GenBank-ID: PZ568891). BhEstB was encoded by a 1461 bp ORF coding for a predicted hydrolase with 486 aa and a predicted molecular weight of 54 kDa. The lack of any secretion signal suggests that the native function of this protein lies within the cell. In comparison with published PET esterases, it shared the highest sequence identity (83%) with BsEstB (GenBank-ID: HM040886) from *B. subtilis* 4P3-11 (Ribitsch et al. 2011). Furthermore, a homolog sharing 74% sequence identity with LipMRD9 (GenBank-ID: WP_034624255.1) from *B. safensis* was found.

### Unique structural features BhEstB apart from bacterial counterparts

**Fig. 1:**
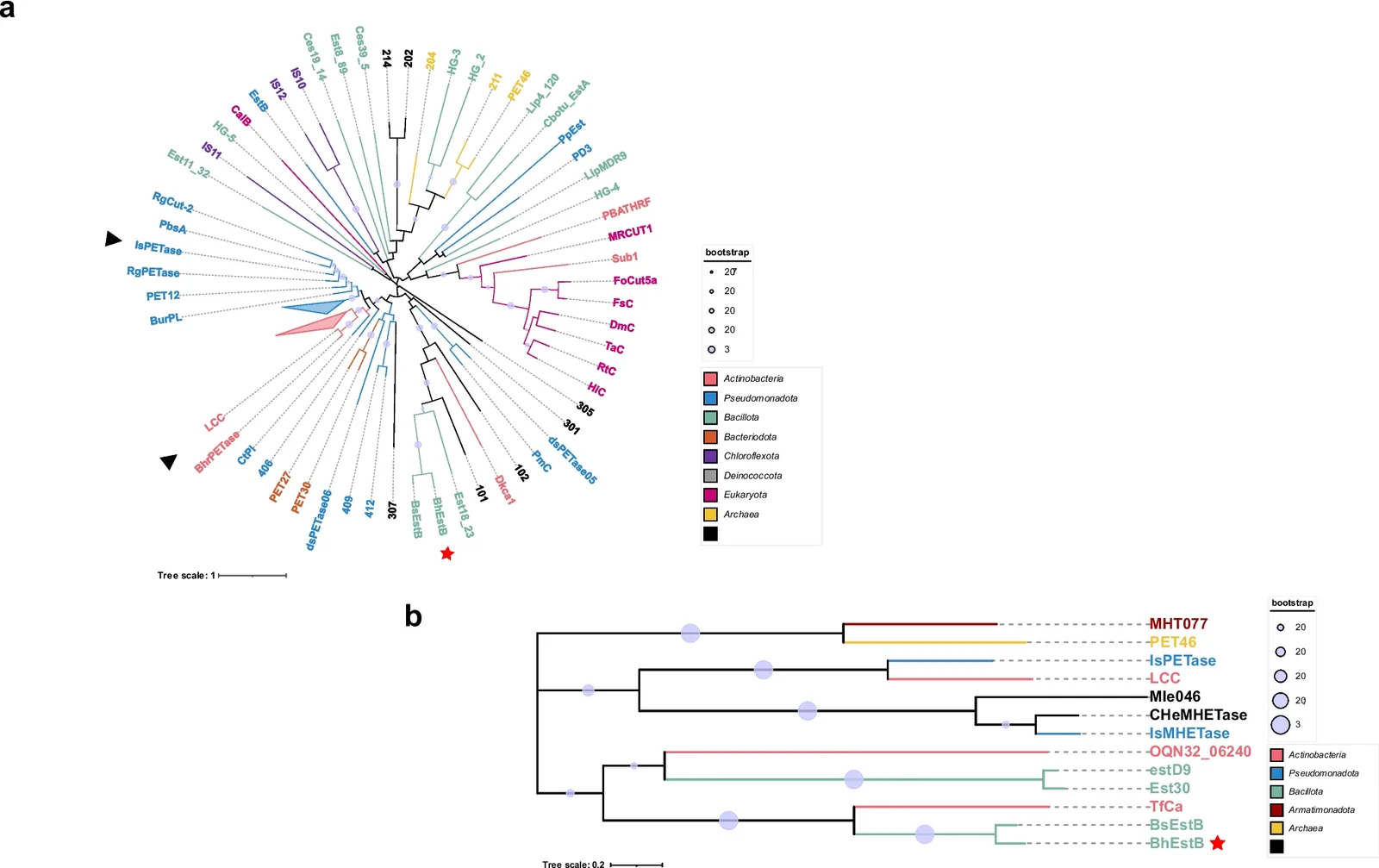
BhEstB exhibits a distinct phylogenetic placement within MHETases compared to PETases. **a** The phylogenetic tree of PET hydrolases, retrieved from the PAZy database in October 2025, identifies two principal clades: *Actinobacteria* (collapsed and shown in blush pink) and *Pseudomonadota* (collapsed and shown in blue). The remaining enzymes are more distantly related and represent a diverse range of bacterial, archaeal, and eukaryotic taxa. BhEstB (highlighted with a star) and BsEstB from *B. subtilis* display high sequence similarity. LCC and IsPETase are marked with a triangle. **b** The phylogenetic tree of MHETases reveals several distinct clusters; notably, BhEstB and BsEstB cluster with TfCa. Both trees were constructed using the maximum-likelihood method, with node support evaluated by 500 bootstrap replicates and represented by node symbols (see legend); only values of 50% or greater are shown. The complete phylogenetic tree from panel **a** is shown in Fig. S1

A further conserved domain (CD) search revealed the presence of a classical carboxylesterase/lipase domain in BhEstB. Its sequence seems to be distant from most bacterial PETases (Fig. 1a and Fig. S1). A sequence-based phylogenetic analysis was conducted to classify published PETases and determine their evolutionary distances and the position of the studied enzyme among them. The 114 analyzed sequences formed several clusters, from close homologs to more diverse sequences. Most sequences cluster within clades related to the bacterial phyla of *Actinobacteria* or *Pseudomonadota*. In contrast, BhEstB clustered closely with BsEstB and was identified as one of the phylogenetically divergent PETases (Fig. 1a). To investigate the ecological occurrence of BhEstB, the global distribution of the *Bacillus subtilis* group—to which *B. halotolerans* belongs—was mapped (Fig. S2) and suggested a global abundance. These findings imply that the enzyme is widespread in ecosystems independent of plastic contamination. Furthermore, a global BLASTp analysis was performed against NCBI’s nr protein database, using a 30% sequence identity and 75% query coverage cutoffs, with *Bacillus* excluded (see Figure_data_Supplementary file). Homologs were found across all bacterial phyla (e.g., TfCa in *Thermobifida fusca*), with the highest similarity to BhEstB observed in *Paenibacillus*. These findings further suggest that these intracellular esterases are widespread in bacteria and likely function in internal metabolism.

The three-dimensional structure of BhEstB was modeled using Alphafold3. It shares the typical fold of α/β-hydrolases with other PETases, composed of a central twisted β-sheet surrounded by α-helices. However, BhEstB contains additional secondary structure elements, including 16 helices and 13 sheets (Fig. S3a). Bulk comparison of BhEstB to a selection of PETase crystal structures (Fig. S4) showed the highest structural similarity to BsEstB. They formed a distinct group alongside enzymes from Bacteria (enzyme 202), Eukaryota (CalB, Hic, FsC, FoCut5a), and Archaea (PET46). Perez-Garcia et al. (2023) also reported that the archaeal PET46 has high structural similarity to feruloyl esterases from Firmicutes, including *Bacillota*. In contrast, known bacterial PETases form a larger, more cohesive cluster with high structural similarity, which is consistent with structure-based alignment results. Additional details on the structure of BhEstB and a comparison of its active site with known PETases are available in (Figs. S3,5 and Supplementary Results S1, Supplementary note 2).

**Fig. 2:**
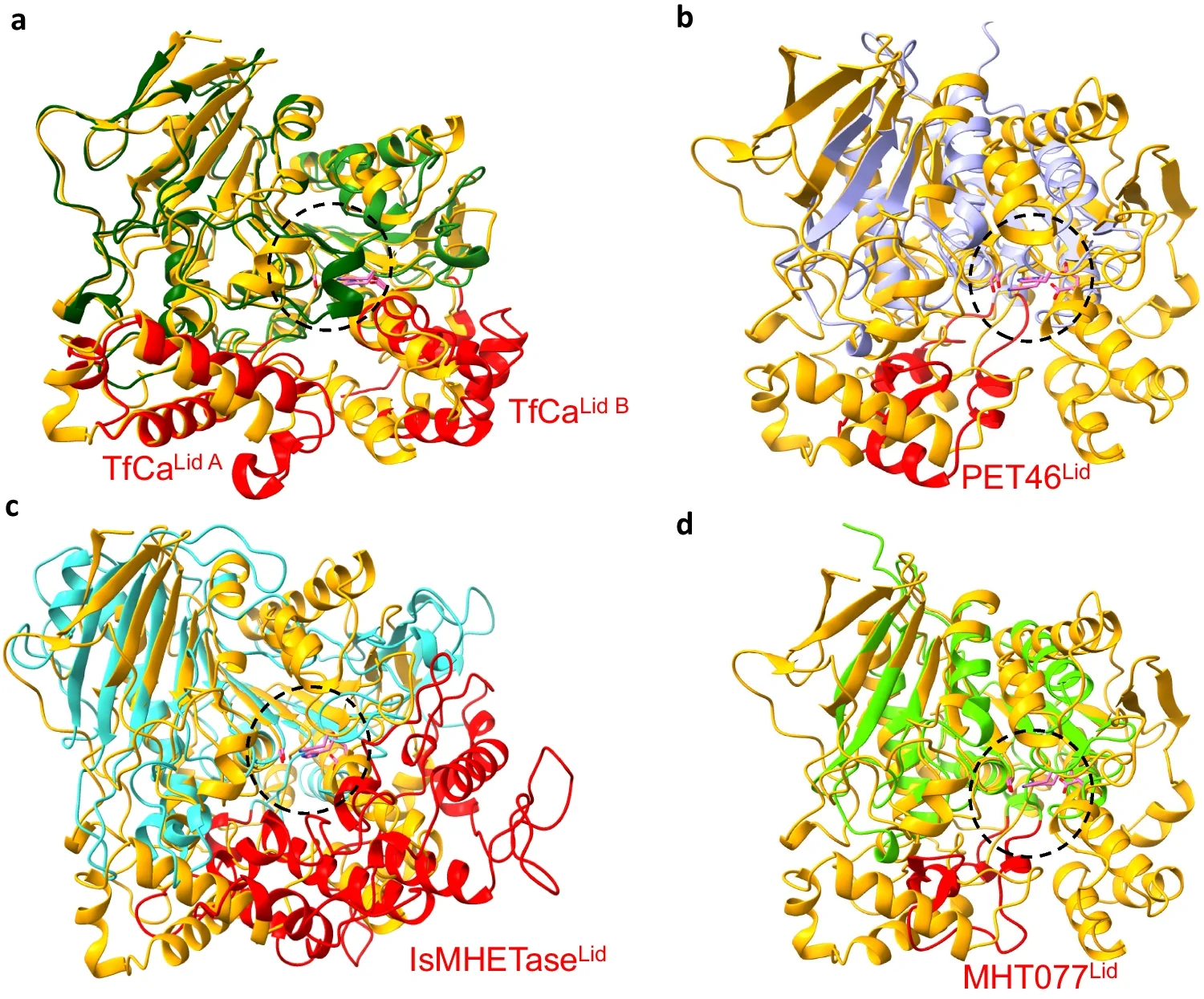
Three-dimensional structural alignment of BhEstB with selected MHETases. BhEstB (amber) and TfCa from *Thermobifida fusca* (dark green) share a similar overall fold (**a**; RMSD = 0.887 Å over 343 pruned Cα atom pairs). Structural alignments with PET46 from Candidatus Bathyarchaeota (lavender blue), IsMHETase from *Ideonella sakaiensis* (turquoise), and MHT077 from Armatimonadota (lime) yielded RMSD values of 1.072, 1.256, and 0.984 Å over 44, 47, and 51 pruned Cα atom pairs, respectively **(b–d)**. Lid domains are highlighted in red. Only TfCa-like lid domains were identified in BhEstB. Active-site regions are indicated by black circles. Detailed substrate-binding groove predictions and active-site comparisons are provided in Figs. S7 and S8

BhEstB was then compared with several known MHETases, as well as LCC and IsPETase, through a phylogenetic analysis (Fig. 1b). Despite the limited number of sequences, the analysis revealed substantial sequence diversity among MHETases, with several distinct groups identified. BhEstB and BsEstB formed a cluster with TfCa from *Thermobifida fusca*, as they all adopt the carboxylesterase fold. The enzymes included originated from diverse organisms and protein families, which supports the observed phylogenetic relationships. BhEstB was then compared individually with selected enzymes to identify shared structural features. Comparison to the original X-ray structure of BsEstB (PDB-ID: 1QE3) showed high structural similarity, particularly in their active sites, which share identical motifs and amino acid compositions (Fig. S6). In contrast, BhEstB and TfCa exhibit lower similarity, sharing only 38% sequence identity, though both maintain a comparable overall esterase fold. BhEstB shows no sequence similarity and is structurally divergent from PET46, MHT077, and IsMHETase. Furthermore, regions homologous to the TfCa lid domains were identified, indicating that the enzyme may have a similar lid architecture. However, no regions homologous to the large lid domain of IsMHETase or the short-lid architecture of PET46 and MHT077 were found (Fig. 2). von Haugwitz et al. (2022) similarly compared TfCa and BsEstB and reported that the *Bacillus* enzyme contains both lid domains characteristic of TfCa. The predicted substrate-binding grooves differed among BhEstB, TfCa, PET46, MHT077, and IsMHETase, although these differences alone do not allow conclusions regarding relative activities (Fig. S7). Notably, the grooves of BhEstB and TfCa were the most similar among the compared enzymes, consistent with their shared carboxylesterase fold. Active site comparison between BhEstB and TfCa revealed identical composition including all residues thought to contribute to MHET binding, whereas the corresponding region of IsMHETase showed substantial differences (Fig. S8). Together, these structural observations imply that BhEstB may also have MHETase activity.

To further assess PET binding within BhEstB’s active site, docking simulations using MHET, BHET, and 3PET were performed (Fig. S9). For each substrate, the orientation with the lowest binding energy was selected: −6.4935 kcal/mol for MHET, −7.2516 kcal/mol for BHET, and −10.3185 kcal/mol for 3PET. In comparison, docking with BsEstB yielded −5.8 kcal/mol for MHET and −6.0 kcal/mol for BHET. In the selected orientations, the reactive carbonyl carbon of the ester moiety was positioned near the catalytic serine, potentially facilitating catalysis. The smallest distances between the substrate’s carbonyl carbon and the hydroxyl oxygen of the catalytic serine were 3.628 Å for MHET, 5.292 Å for BHET, and 7.111 Å for 3PET.

### BhEstB hydrolyses pNP-esters and BHET at mesophilic temperatures

Following its sequence-based characterization, biochemical characterization of BhEstB was performed. The heterologous expression as 6-His-tagged protein consistently yielded high amounts (40–60 mg/L) of ~98% pure protein after His-tag purification (Fig. S10), showing its ease of expression and excellent solubility.

**Fig. 3:**
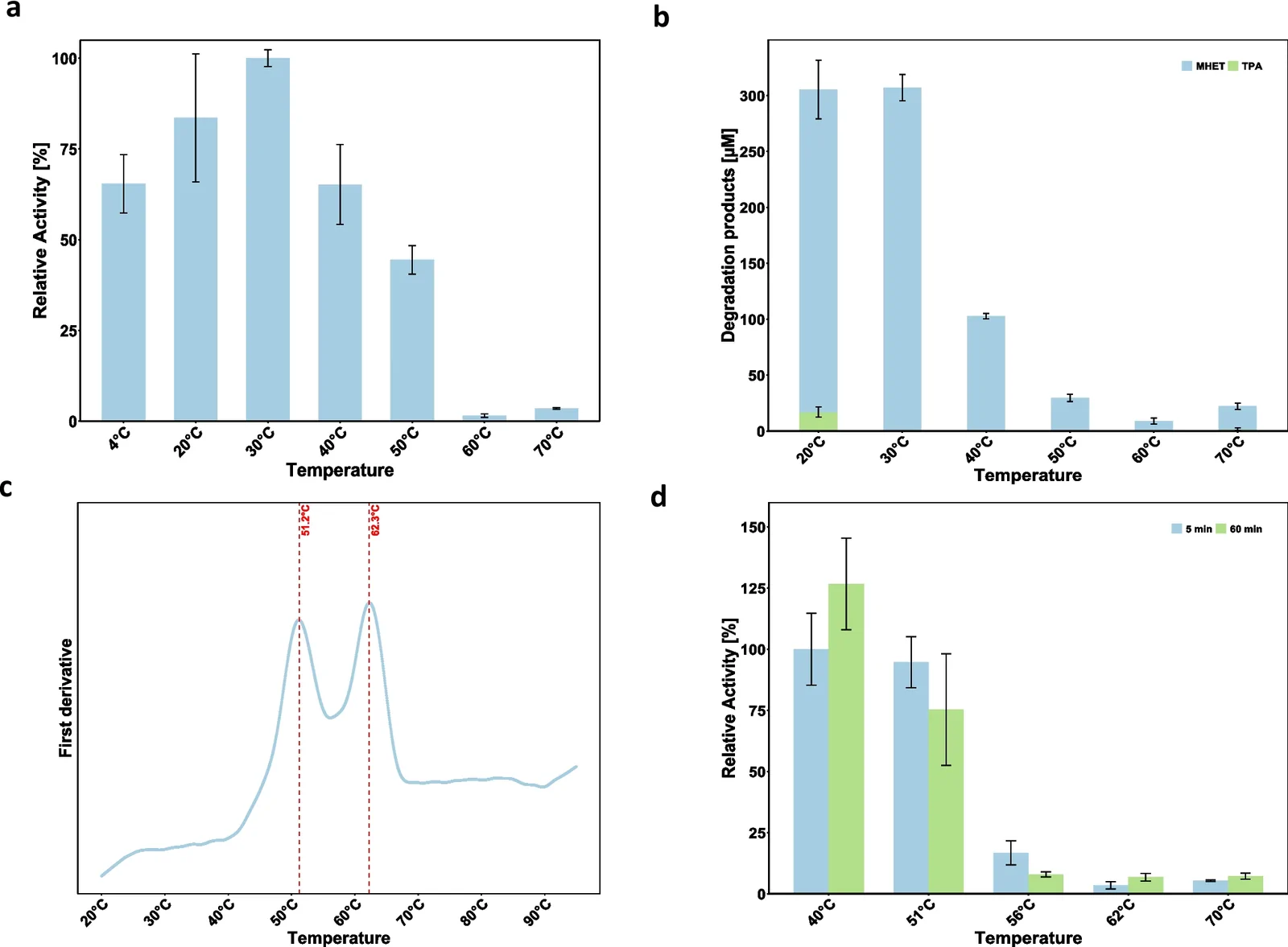
Thermal tests show that BhEstB stays active up to 50 °C. **a**, **b** BhEstB activity was evaluated using pNP-ester C10 (decanoate) and BHET as substrates, respectively. The enzyme displayed a broad maximum activity plateau between 20 and 30 °C for both assays. Activity decreased sharply for BHET above 40 °C and for pNP-ester C10 above 60 °C. **c** NanoDSF analysis revealed two thermal transitions at 51.2 °C and 62.3 °C, consistent with a multi-step or biphasic unfolding process. **d** pNP-esterase activity at 5 and 60 min showed enzyme activation at 40 °C, about a 25% loss at 51 °C after 1 h, and a pronounced loss at 56 °C within 5 min. These results collectively establish both the temperature optimum and thermal stability limits of BhEstB

BhEstB showed no activity on long-chain *p*NP esters (C16–C18), confirming its classification as an esterase, and regarding pH characterization, the highest activity was observed at pH 8 (Fig. S11). The enzyme was active between 4 and 50 °C, with maximum activity at 20–30 °C and a sharp decline above 60 °C. The activity against BHET was tested across the same temperature range. Activity dropped significantly above 40 °C. This may in part be due to BHET autohydrolysis at high temperatures, which reduces apparent enzyme activity (Fig. 3a, b). Residual activity was consistently detected at high temperatures with both BHET and *p*NP substrates.

To evaluate thermal stability, BhEstB’s melting temperature was determined using NanoDSF. Two T_m_ values were identified: 51.2 °C and 62.3 °C (Fig. 3c). This profile is consistent with a multi-step unfolding process, which has been reported for modular or multidomain proteins and may reflect structurally distinct conformational transitions during thermal denaturation. (Magnusson et al. 2019). However, the molecular origin of the observed transitions cannot be conclusively determined from the present dataset alone. To test this, BhEstB was incubated at five temperatures for 5 and 60 min, followed by activity measurement using *p*NP C10. After 1 h, full activity was retained at 40 °C, whereas activity decreased by 25% at 51 °C. At temperatures above 56 °C, more than 90% of activity was lost, even after only 5 min (Fig. 3d). These results indicate BhEstB remains active up to 50 °C, but loses function rapidly above this point. The absolute melting temperature is likely 62.3 °C.

### BhEstB is active on 3PET and degrades BHET and MHET at high rates

To further assess BhEstB’s potential for PET degradation, we characterized its catalytic activity toward PET intermediates (3PET, BHET, and MHET) under various conditions. Assays were performed alongside benchmark enzymes using identical experimental setups. LCC^ICCG^ was selected as an industrially optimized PETase, while IsMHETase and PET46 were included for their high activity on MHET and BHET, respectively. Degradation of 3PET was first assessed. BhEstB degraded > 98% of the substrate within 24 h at 30 °C, whereas a substantial loss of activity was observed at 60 °C. As for the benchmark enzymes, IsMHETase showed no trimer-degrading activity in 24 h, whereas LCC^ICCG^ and PET46 exhibited slightly higher degradation rates than BhEstB (Fig. S11).

**Fig. 4:**
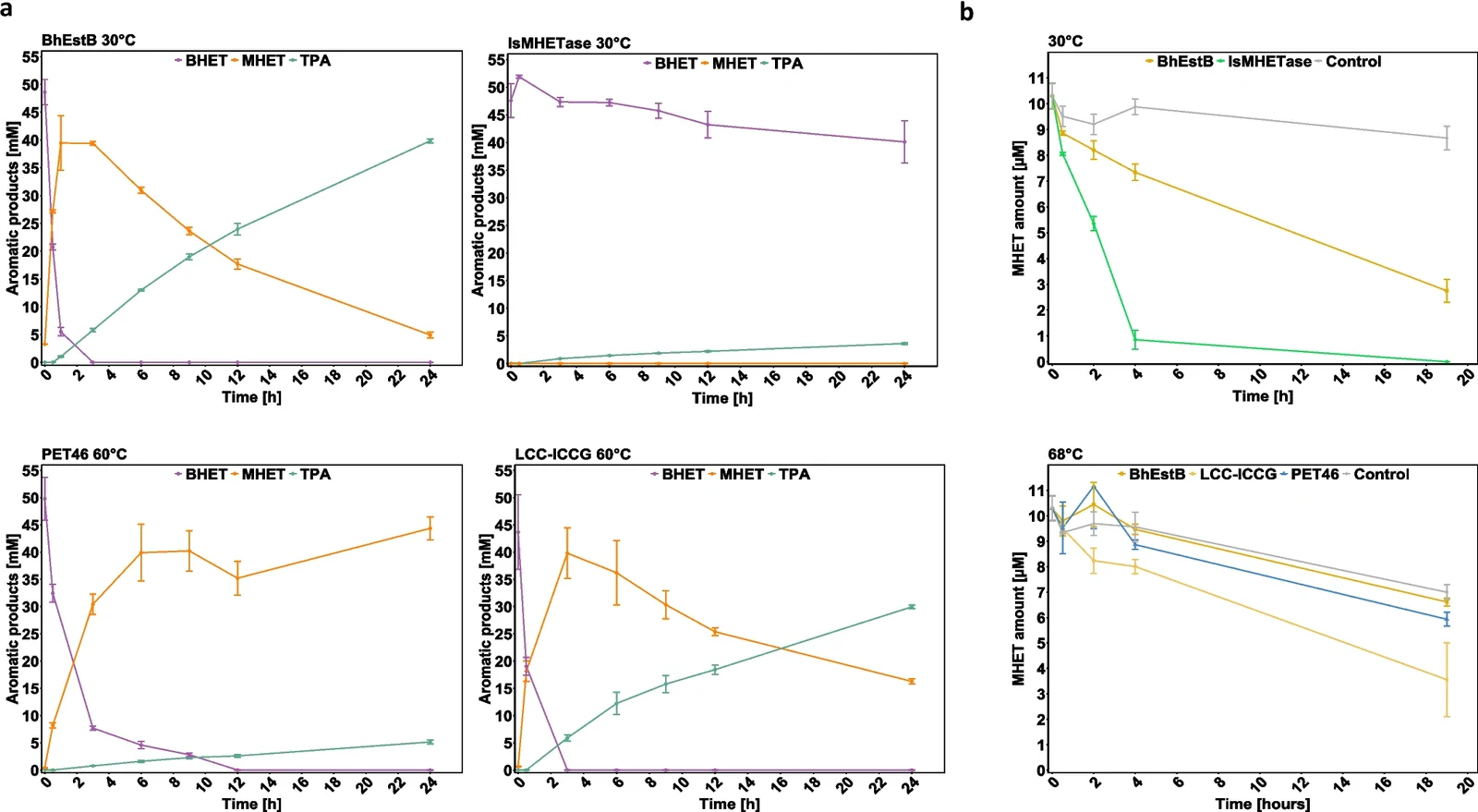
BhEstB is a highly active BHETase, and degrades MHET better than PET46 and LCC-ICCG. **a** Degradation kinetics of 50 mM BHET over 24 h at various temperatures, comparing BhEstB to benchmark enzymes. Under these conditions, BhEstB degraded both BHET and MHET more rapidly than all other enzymes tested. **b** Degradation of 10 mM MHET by various enzymes over 20 h at different temperatures. The time courses display MHET depletion, with BhEstB achieving 70% degradation and IsMHETase reducing MHET to 0 µM. MHET was generated by a 4-h PET powder degradation using LCC-ICCG. Controls without added enzyme were included at each temperature. For all panels, data represent the average of three independent replicates. Additional plots for both assays are provided in Figs. S13, S14 and S16

Further, the enzymatic activity of BhEstB toward BHET was evaluated under various conditions. Interestingly, the enzyme degraded 50 mM of BHET in just 1 h at 30 °C and converted 39.8 mM of MHET (77% ± 0.7%) into TPA in 24 h, indicating excellent BHETase activity. Furthermore, it retained good activity against BHET even at 4 °C and completely degraded 50 mM in a time period of 6 h. Notably, a significant decline in MHETase activity was observed at 4 °C. Whereas at 60 °C, no difference was observed and compared to the negative control, in which BHET was degraded due to high temperature (Fig. 4a, Figs. S13 and S14). No BHETase activity was observed from the IsMHETase; however, it degraded all MHET released from autohydrolysis of BHET at 30 °C. At 60 °C, LCC^ICCG^ degraded 50 mM BHET in 3 h and degraded up to 67% (± 0.7%) of MHET in 24 h. PET46 degraded BHET at a slightly slower rate (12 h) and did not show significant MHETase activity (10%) (Fig. 4a).

### BhEstB efficiently degrades MHET in PET degradation cascades

Previous assays demonstrated that BhEstB degrades a substantial amount of MHET, outperforming both LCC^ICCG^ and PET46. However, direct comparison with IsMHETase was not feasible because IsMHETase is inactive on BHET. Therefore, further characterization using MHET was necessary to more accurately benchmark the MHETase activity of BhEstB. Given the high cost and limited availability of MHET at larger scales, and to mimic the PET degradation mixture, LCC^ICCG^ was incubated for 4 h with PET powder to allow the accumulation of MHET. The 4-h incubation of PET with active LCC^ICCG^ yielded around 20 mM of aromatic products, of which 50% were usually MHET (Fig. S15). Prolonged incubation until 24 h yielded 20% more products, with 87% TPA. The release of MHET has been demonstrated to slow PET degradation, as it binds in a non-catalytic pose to the active site of PETase (Zhang et al. 2023).

To overcome this bottleneck, MHET degradation was performed from the supernatant in a separate setup, and the powder was incubated again in the fresh buffer. BhEstB and IsMHETase were tested at several temperatures, while LCC^ICCG^ and PET46 were tested at 68 °C (Fig. 4b and Fig. S16). MHET was most efficiently degraded by IsMHETase under all tested temperatures, achieving complete conversion to TPA within 4 h at 30°°C, 40°°C, and 50 °C. BhEstB exhibited optimal activity toward MHET at 30 °C by degrading 73% (± 4%) within 20 h. Except for IsMHETase, none of the tested conditions resulted in better performance than that of any of the enzymes, including LCC^ICCG^. Although BhEstB exhibited slower MHET hydrolysis compared to the specialized IsMHETase, it outperformed again both PET46 and LCC^ICCG^, whose degradation is enhanced by elevated temperature.

Table 2 summarizes enzymes acting on PET, BHET, and MHET. Carboxylesterases demonstrate potential for intermediate degradation; however, with respect to PET degradation, their primary limitation is lack of thermal stability. This is contrary to PET46 and MHT077, which exhibit greater thermostability, but moderate to low activities on BHET/MHET. IsMHETase does not act on the polymer or BHET and lacks thermostability. In comparison, LCC^ICCG^ demonstrates relatively high thermostability, high activity on PET and BHET, and efficient MHET degradation during extended incubations. Despite the aforementioned limitations, several enzymes have been shown to enhance PET degradation yield and TPA production when combined with a PET hydrolase, including IsMHETase, BsEstB, MHT077, and TfCa (Knott et al. 2020; von Haugwitz et al. 2022; Li et al. 2023; Murphy et al. 2026).

**Table 2:** Qualitative* comparison of esterase and cutinase activity toward PET, BHET, and MHET, as well as thermal stability (T_m_)

	MHET activity	BHET activity	PET activity	T_m_ (°C)	Reference
BhEstB	High	Very high	Low	62.3	This work
IsMHETase	Very high	None	None	50	This work, (Sagong et al. 2020)
LCC^ICCG^	Moderate	High	Very high	94.5	This work, (Tournier et al. 2020)
PET46	Low	Low	Low	84.55	This work, (Perez-Garcia et al. 2023)
BsEst	High	Very high	None	-	(Li et al. 2023)
TfCa	High	Very high	None	64.5	(von Haugwitz et al. 2022)
MHT077	Moderate	-	-	94.6	(Murphy et al. 2026)

^*^Activity ratings are qualitative descriptors based on data obtained from this work and the literature under different experimental conditions and are therefore not directly quantitatively comparable across enzymes

### BhEstB exhibits efficient BHET hydrolysis

**Fig. 5:**
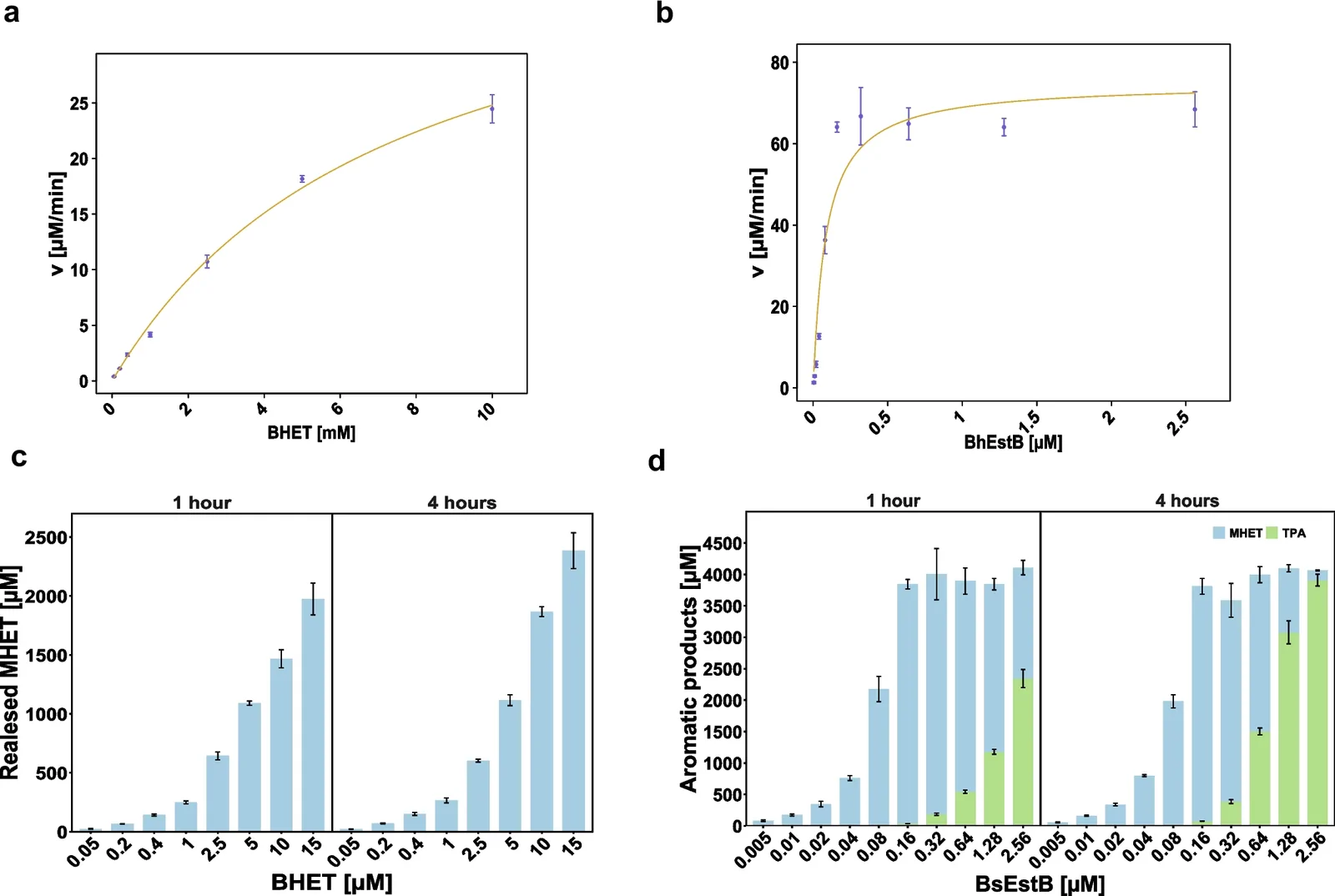
Raw and fitted data from conventional and inverse Michaelis–Menten analyses of BHET hydrolysis by BhEstB. **a** and **c** display fitted kinetic curves of BHET hydrolysis (1-h data) for conventional and inverse Michaelis–Menten kinetics, respectively. The conventional Michaelis–Menten experiment was performed using a constant enzyme concentration of 0.04 µM, whereas the inverse Michaelis–Menten experiment was conducted at a fixed BHET concentration of 5 mM. In all experiments, BHET was dissolved in phosphate buffer pH 8. The fitted curve was generated using a nonlinear regression analysis in R (v.4.4.1). The kinetic parameters extracted from these fits may be found in Table 3. **c** and **d** show the release of aromatic products at 1 and 4 h from conventional and inverse kinetic experiments, respectively. All experiments were conducted in triplicate at 30 °C, with an enzyme-free control for each condition. Data points represent mean initial reaction rates with error bars (± SD)

After observing that BhEstB outperformed the other enzymes on BHET, both conventional and inverse Michaelis–Menten kinetics were next applied to gain deeper insights into its catalytic performance for BHET. The enzyme exhibited a high K_m_ (6.54 ± 1.01 mM) and K_cat_ (15.57 ± 1.25 s^**−1**^), indicating a moderate affinity for BHET, and a good turnover rate (Fig. 5a). In comparison to its homologs from *B. subtilis,* BhEstB showed lower affinity for BHET, and a comparable turnover rate (Table 3). Nevertheless, the authors reported that the activity of both homologs on BHET was mostly similar (Bååth et al. 2021; Li et al. 2023). A variation in the BHET breakdown kinetic parameters by IsPETase was also observed, although the differences were less pronounced (Bååth et al. 2021; Liu et al. 2022). Despite variations in the reported data and experimental setups, it was confirmed that in all studies, BhEstB or its homologs from *B. subtilis* exhibit higher catalytic efficiency for BHET than the other enzymes.

**Table 3:** Kinetic parameters for BHET hydrolysis by BhEstB and benchmark enzymes. BhEstB data represent mean values from three replicates. Data for the other enzymes were obtained from the cited references

Enzyme	K_m_ (mM)	K_cat_ (s^−1^)	K_cat_/K_m_ (M^−1^⋅ s^−1^)	Reference
BhEstB	6.54 ± 1.01	15.57 ± 1.25	(2.38 ± 0.42) × 10^3^	This work
BsEst*	0.1 ± 0.02	37.7 ± 1.98	(382.11 ± 84.51) × 10^3^	Li et al. (2023)
BsEst*	0.77 ± 0.08	13 ± 0.80	(17 ± 2.1) × 10^3^	Bååth et al. (2021)
HiC	3.7 ± 0.24	2.0 ± 0.09	(0.55 ± 0.04) × 10^3^	Bååth et al. (2021)
TfC	0.83 ± 0.31	1.6 ± 0.26	(1.9 ± 0.76) × 10^3^	Bååth et al. (2021)
IsPETase	1.4 ± 0.08	4.8 ± 0.15	(3.3 ± 0.21) × 10^3^	Bååth et al. (2021)
IsPETase	4.48 ± 0.54	5.95 ± 0.62	(1.33 ± 0.02) × 10^3^	Liu et al. (2022)
MtCut	3.07 ± 0.93	4.27 ± 1.15	(1.41 ± 0.07) × 10^3^	Liu et al. (2022)
LCC^ICCG^	2.58 ± 0.98	2.29 ± 0.95	(1.00 ± 0.17) × 10^3^	Liu et al. (2022)
IsMHETase	0.91	0.02	0.03 × 10^3^	Sagong et al. (2020)

^*^Two homologs of a PET esterase from *B. subtilis* exhibiting 98% identity

It is interesting to mention that BHET is insoluble in water, especially at high concentrations, which may be a significant contributor to the divergence of the observed results; in this case, the solvent used and the substrate concentration range greatly influence the outcomes. For instance, some studies used an organic solvent to improve BHET solubility (von Haugwitz et al. 2022). Since BHET is poorly soluble in water, we applied inverse Michaelis–Menten kinetics to determine the maximal turnover rate and substrate affinity. BhEstB degraded BHET starting from a low concentration (0.005 µM) and reached maximum velocity starting from 0.16 µM, with a notably lower _inv_K_m_ value (0.1 ± 0.02 mM) (Fig. 5b, Table 3). These results are consistent with efficient interfacial turnover under partially insoluble reaction conditions and suggest that BHET breakdown in this case likely follows the insoluble substrate model (Kari et al. 2017; Andersen et al. 2018). Moreover, between the 1- and 4-h results (Fig. 5c, d), it was observed that at enzyme concentrations up to 0.16 µM (including the concentration used for the conventional kinetics experiment), no significant difference was observed, suggesting that enzyme deactivation may contribute to the reduced activity. Furthermore, higher enzyme concentrations were required to degrade MHET; as mentioned above, 0.16 µM was sufficient to degrade 4 mM BHET in only 1 h, whereas for MHET, significant degradation was observed only from 0.64 µM and after 4 h.

### BhEstB is active on PET powder at elevated temperatures

**Fig. 6:**
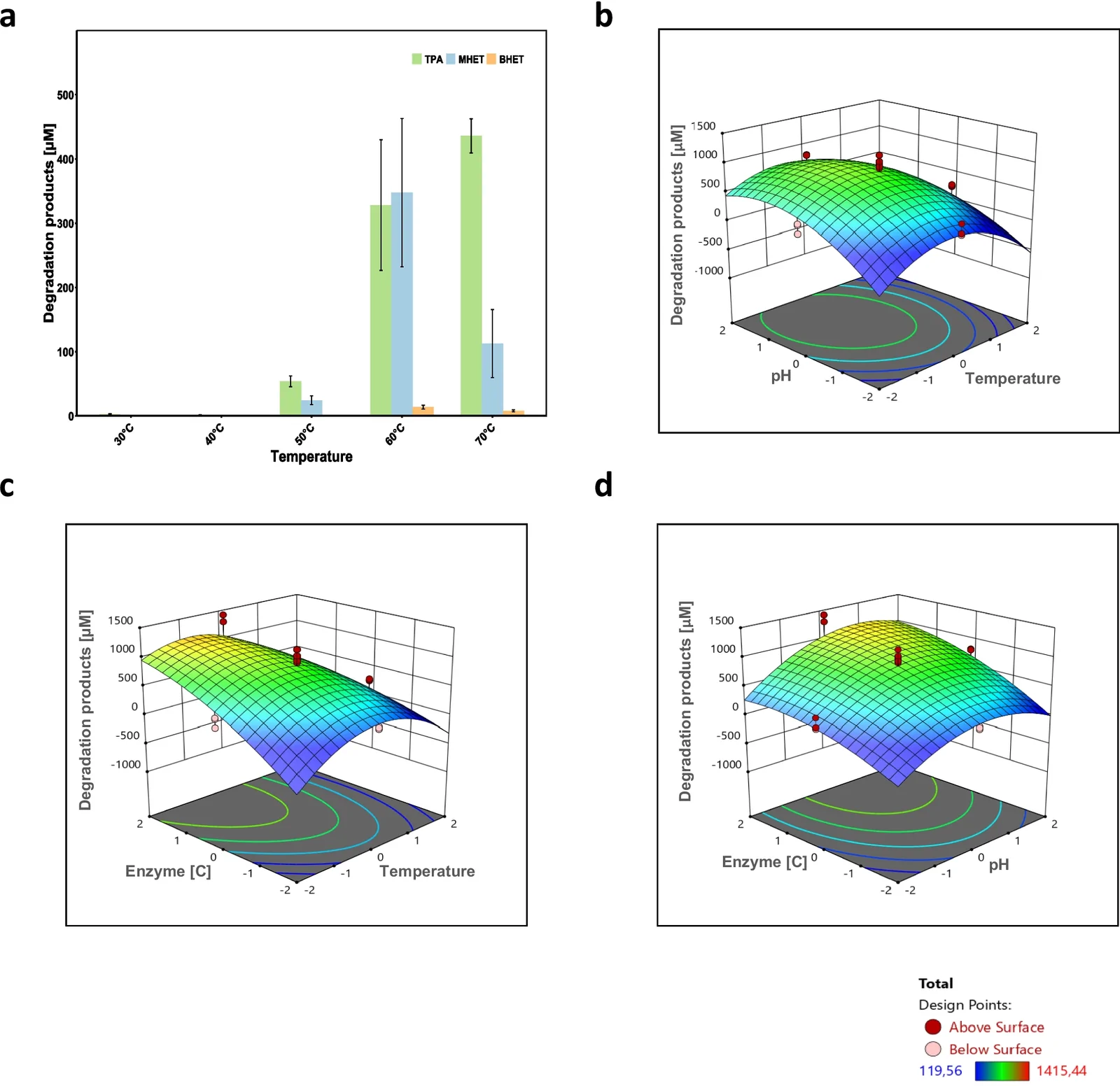
BhEstB degrades PET at elevated temperatures. **a** Twenty-four hours of incubation demonstrated that BhEstB degrades the tested PET powder at temperatures starting from 50 °C, with a higher amount of released degradation products at 60 °C, and a better conversion of MHET into TPA at 70 °C. Activity was significantly higher than in the corresponding enzyme-free controls under all active conditions tested (*p* < 0.01). **b**, **c**, and **d** Three-dimensional surface plots of 2-factor interactions influencing PET degradation by BhEstB, showing that optimal activity is achieved at the highest enzyme concentration (panels **c** and** d**), at a pH above the central level (pH 8) (panels** b** and** d**), and at temperatures under 65 °C (panels **b** and** c**). The corresponding contour plots are shown in Fig. S16

Finally, BhEstB was tested on PET amorphous film and semi-crystalline powder, alongside benchmark enzymes. No activity was detected on PET film, but degradation was observed with powder, with BhEstB showing higher activity at 60 °C and 70 °C, producing 689.50 µM and 556.37 µM of total aromatic products (TPA, MHET, BHET), respectively (Fig. 6a). For comparison, the activity of IsPETase and LCC was tested under identical conditions at their respective optimal temperatures (Table S1).

### Modeling verifies biochemical traits by using central composite design (CCD) and artificial neural networks (ANN)

Within this framework, a hybrid optimization approach was employed to better understand and integrate the complex factors influencing PET activity, specifically temperature, pH, and enzyme concentration. Initially, a design of experiments (DoE) method using CCD was implemented to test various combinations (Table S2). The significance of the model and factors was subsequently evaluated through analysis of variance (ANOVA) as part of the RSM, as described by Guendouzi et al. (2024). The *F*-value of the model is 15.02, which exceeds the tabulated value of 2.13, indicating that the model is statistically significant. Furthermore, the model has a *P*-value of 0.000, confirming its adequacy and statistical significance (Table S3). However, the lack-of-fit test yielded a *P*-value of 0.000, implying that the model exhibits a statistically significant lack of fit. This suggests the model may not adequately represent the data; hence, further refinement or an alternative modeling approach may improve its predictive accuracy (Alloun et al. 2023). Further 3D surface plots were generated to better visualize the response landscape and identify optimal conditions. Notably, degradation was always highest when the BhEstB concentration was at its maximum, whereas for temperature, the optimum was always observed at temperatures above 65 °C; lastly, pH above the central level (8) yielded more degradation products (Fig. 6b–d).

Given that the RSM approach did not provide sufficient accuracy in modeling and predicting PET degradation, an ANN-based nonlinear regression was implemented to improve prediction, modeling, and optimization (see supplementary results S2, Fig. S17-S21). Subsequently, a genetic algorithm was applied for optimization. The predicted maximum concentration of released products was 1286 µM, while the experimentally measured value was slightly lower at 1260 µM (Table S4). The absolute error between the predicted and observed values was 2%, indicating strong agreement between the genetic algorithm optimization and the experimental results for total degradation product release.

## Discussion

In the present study, a PET-active enzyme (BhEstB) was identified from *B. halotolerans* HGR5 using an HMM-based screening, and the recombinant enzyme was characterized. It is well known that many PET-degrading enzymes have been discovered using HMMs and similar sequence-based bioinformatics approaches. These methods identify conserved motifs across diverse ORFs, enabling the discovery of highly active and distinct PETases, which might not have been found with conventional homology-based methods (Danso et al. 2018; Erickson et al. 2022).

The closest homolog of BhEstB is BsEstB from *Bacillus subtilis* (Li et al. 2023). In their study, Li et al. showed that BsEstB retained 70% activity at 60 °C and 25% at 70 °C, indicating improved thermotolerance compared to most mesophilic *Bacillus* enzymes (Vieille and Zeikus 2001). BsEstB exhibited activity on long-chain pNP (C16) and olive oil, but not on PET foil; activity on powder was not assessed. Notable degradation of BHET and MHET by BsEstB was observed at 30 °C, although the BHET concentration tested was one-tenth of that used in this study and a direct comparison with IsMHETase has not been conducted (Perez-Garcia et al. 2023). Perez-Garcia et al. (2023) reported similar results regarding 3PET degradation by PET46 but observed rapid hydrolysis of BHET at a low enzyme concentration, indicating both good substrate affinity and turnover rate; nonetheless, high substrate levels were not investigated, leaving the substrate saturation behavior unknown. Sagong et al. reported that IsMHETase can degrade both BHET and PET pentamer (5PET). However, the study further noted that the wildtype MHETase exhibits limited Exo-PETase and BHETase activities (Sagong et al. 2020). (von Haugwitz et al. 2022) reported that TfCa exhibits high activity toward both BHET and MHET, with BHET being degraded more efficiently. Molecular dynamics simulations revealed that TfCa has a lower binding affinity for MHET than IsMHETase. Collectively, these findings indicate that although BhEstB and related carboxylesterases demonstrate higher activity toward BHET, they also exhibit substantial activity against MHET. The native function of EstB in *Bacillus* has not been elucidated. However, previous works have reported its activity against *p*-nitrobenzyl (PNB) esters, *β*-lactam antibiotics (e.g., loracarbef, cephalexin) by targeting their ester bond; it also displayed secondary amidase activity (Chen et al. 1995; Galmés et al. 2021). These findings suggest a general broad substrate specificity toward various aromatic esters, and this may explain the activities observed in this study against BHET and MHET, independent of prior exposure to PET or its intermediates.

For PET esterases, optimal PET activity is assumed to occur at about 10 °C above the soluble substrate optima, due to improved substrate accessibility (Wei et al. 2022; Pérez-García et al. 2025). This study shows that BhEstB exhibited optimal activity on the tested semi-crystalline PET at 60 °C. An apparent contradiction exists between BhEstB’s thermal stability profile and the product release observed from PET powder at 60–70 °C. Since time-resolved product release and residual activity measurements were not performed, the present data do not allow mechanistic resolution of this behavior. One possible explanation is that BhEstB remains transiently active during the early stages of incubation, enabling hydrolysis of highly accessible substrate regions, such as amorphous surface domains or oligomeric material potentially present in the PET powder, before progressive thermal inactivation occurs. IsPETase also exhibited optimal activity on PET at 40 °C, although its reported melting temperature ranges from 40 to 50 °C depending on buffer and protocol (Meng et al. 2021; Rennison et al. 2021; Liu et al. 2023). Similarly, IsMHETase showed excellent activity at 50 °C, close to its reported Tₘ. It was also observed elsewhere that co-immobilized IsMHETase with DuraPETase showed a high MHET conversion from 30 to 50 °C (Chen et al. 2022). Likewise, some engineered PETase variants also exhibit optimal activity near their thermal stability limits (Stevensen et al. 2025). Wu et al. (2025) observed low correlations between enzymatic activities and T_m_ for PETases, proving that PETase activity is complex and influenced by multiple factors. Collectively, these examples provide a possible explanation for the observed activity of BhEstB toward PET near its melting temperature.

The applied optimization model provided insight into PET degradation by BhEstB. Additionally, nonlinear modeling through ANN explained the data better, enabling improved understanding of each factor’s influence on the degradation. However, it appears that the optimal enzyme concentration was not captured within the tested range. A refinement of the model could have addressed this, but the analysis was limited to the initial model, since the enzyme concentrations at the upper range were already high, and further increases would not be practical for large-scale applications. The observed results could be explained by the thermolabile nature of BhEstB, as mentioned above. In addition, considerable variability between different enzyme batches was observed, with some showing up to fivefold less degradation products. Given these challenges, it is evident that BhEstB will not be used for PET degradation per se, but in combination with highly active enzymes or in engineered strains, it may be a helpful tool to improve industrial polymer recycling and make it more efficient. Further improvement of the enzymes’ thermal stability may enhance activity against the polymer; however, this might be a very long road and will not guarantee performance comparable to PET-adapted enzymes like LCC^ICCG^ or variants of the IsPETase (Tournier et al. 2020; Lu et al. 2022; Bell et al. 2022).

Research on the molecular evolution and global distribution of enzymes involved in plastic degradation is an emerging field. Recent work established that PET hydrolases can be found across phylogenetically diverse organisms and habitats (e.g., landfills, human saliva metagenomes) (see PAZy.org). Given that plastic pollution is a recent phenomenon (< 70 years), most of these activities only recently evolved or perhaps unselected side activities of versatile hydrolases (Danso et al. 2019; Wei et al. 2025). This rationale applies most likely to all divergent PETase. While BhEstB is only relatively poorly active on the PET polymer, it degrades BHET and MHET very efficiently.

The current study enriched our knowledge on BHET- and in part PET-active enzymes from the *Bacillus* genus, demonstrating their widespread potential to degrade BHET and MHET (in large quantities), and in part PET. Furthermore, the kinetic data offer practical guidance for characterizing and engineering PETases, BHETases, and MHETases to improve PET degradation efficiency. While the main focus of the present work was enzyme characterization, it would be valuable to investigate the strain’s potential in PET degradation and to identify the enzymes and pathways possibly involved.

## Supplementary Information

Below is the link to the electronic supplementary material.

ESM 1 PDF (3.24 MB)

## Data Availability

No datasets were generated or analysed during the current study.
