# Supplementary material for: A versatile Bacillus esterase reveals a carboxylesterase blueprint for PET intermediate degradation
Source: Appl Microbiol Biotechnol. 2026 Oct 9;110:304. doi: 10.1007/s00253-026-14056-w (PMC13650222; doi:10.1007/s00253-026-14056-w)
Supplement: Supplementary file 1 [file 253_2026_14056_MOESM1_ESM.pdf]

A Versatile Bacillus Esterase Reveals a Carboxylesterase Blueprint for PET Intermediate Degradation

Hadjira Bounabi[1,2], Bilal Yahiaoui[3], Meriem Gasmi[2], Meroua Safa Mechouche[2], Nico Bäse[1], Marno Gurschke[1], Rebecka Molitor[4], Pablo Pérez-García[1] and Wolfgang R. Streit[1,*]

[1]Department of Microbiology and Biotechnology, University of Hamburg, Ohnhorststrasse 18, 22609 Hamburg, Germany
[2]Biotechnology Laboratory, Higher National School of Biotechnology Taoufik Khaznadar, Nouveau Pôle Universitaire Ali Mendjeli, Constantine 25100, Algeria
[3]Laboratory of Applied Microbiology, Department of Microbiology, Faculty of Natural and Life Sciences, Ferhat Abbas University, Setif 19000, Algeria
[4]Institute of Molecular Enzyme Technology, Heinrich-Heine-University Düsseldorf, Forschungszentrum Jülich, Jülich, Germany

*Corresponding author: Prof. Dr. Wolfgang R. Streit
Adresse: Institute of Plant Sciences and Microbiology, Department of Microbiology and Biotechnology, University of Hamburg, Ohnhorststraße 18, 22609 Hamburg, Germany
Email : wolfgang.streit@uni-hamburg.de

SUPPLEMENTARY RESULTS :

S1: In silico characterization of BhEstB

BhEstB shares the overall α/β-hydrolases fold with other PETases, but also harbors extra 2D elements (**Fig. S3**). Unique structural elements include helices α1, α5–α7, α10–α16 and sheets β1, β2, β4, and β13. The catalytic triad comprises Glu310, His399, and Ser189, making BhEstB one of the few PET-degrading enzymes with glutamate instead of aspartate in the active site. The active site entrance loops were identified as residues 64–71 and 413–417 on one side, and 316–320 and 260–268 on the other, consistent with previous structural studies of BsEst (Spiller et al. 1999). According to a ConSurf analysis, the additional secondary structure features in BhEstB are located in variable regions. According to the DoGSiteScorer prediction of the Protein+ server, the surface area and volume of the active site pocket are 806.92 A2 and 726.66 A3, respectively.

Structural alignment revealed that the predicted BhEstB's binding site architecture differs significantly from those of LCC, IsPETase, and PHL-7 (**Fig. S5)**. BhEstB lacks the Trp-Met-Tyr/Phe motif known to facilitate PET binding. In LCC, IsPETase, and PHL-7, a methionine follows the catalytic serine and contributes to the oxyanion hole; in BhEstB, this methionine is replaced by alanine, with a methionine appearing three residues downstream at position 193. Additionally, BhEstB features an alanine at position 107 (instead of tyrosine or phenylalanine, with nearby aromatic residues at positions 108 and 109. Also, the conserved tryptophan forming the aromatic clamp in other PETases is replaced by serine, with no compensatory aromatic residues nearby. Furthermore, the third aromatic clamp component (isoleucine or valine in other PETases) is absent in BhEstB, which instead has a methionine at the corresponding position. (Magalhães et al. 2021; Liu et al. 2023)

To explore potential substrate-binding interactions, aromatic residues surrounding BhEstB's active site were examined. Several of these residues are located within unique secondary

structure motifs and lack direct counterparts in other PETases. Tyr70, Trp102, Tyr118, Phe186, and Phe407 were identified as forming an aromatic clamp, potentially aiding substrate binding. Tyr118 is predicted to form a hydrogen bond with MHET. Additional residues, including Phe314, Phe115 (three residues downstream of Glu310), and Phe363 are positioned near the active site with their aromatic rings oriented toward it. Phe271 and Phe315 are predicted to form hydrogen bonds with BHET and 3PET, although their side chains are not perfectly aligned. These findings suggest BhEstB may utilize a distinct set of aromatic residues for substrate binding. BhEstB's homolog has previously been compared to human acetylcholinesterase (AChE) and is used as a model for studying mammalian carboxylesterases (Spiller et al. 1999; Streit et al. 2008). A BLASTp search revealed 33% sequence similarity (96% query coverage), and structural alignment showed that both enzymes share a similar overall fold (**Fig. S6**).

S2 : Optimization of PET activity

To optimize PET degradation activity by BhEstB, temperature (X1), pH(X2) and enzyme concentration (X3) were selected as variables, since they affect PET depolymerization greatly. The quadratic equation (**equation 2**) derived from the model and the results were used to determine the relation of each factor or interaction and the response.

$$Y = 856.0 - 123.8 \times X1 + 123.8 \times X2 + 191.3 \times X3 - \\ - 175.2 \times X1X1 \; - 109.9 \times X2X2 \\ -48.5 \times X3X3 - 33.0 \times X1X2 - 71.9 \times X1X3 + \\ 29.5 \times X2X3 \qquad (2)$$

To validate the model, its F-value must exceed the critical value from the F-distribution for the given degrees of freedom and a level of significance of α = 5%, and its P-value must be less than 0.05 (Guendouzi et al. 2024). The F-value of the model is 15.02, which exceeds the tabulated value of 2.13, indicating that the model is statistically significant. Furthermore,

the model has a P-value of 0.000, confirming its adequacy and statistical significance (**Table S2**). Additionally, the coefficient of determination (R^2) is 0.7544 indicating that approximately 75.44% of the variability in the response can be explained by the chosen levels of the input variables. However, the lack-of-fit test yielded a P-value of 0.000, implying that the model exhibits a statistically significant lack of fit (Ahmad and Alrozi 2010). This suggests the model may not adequately represent the data; hence, further refinement or an alternative modeling approach may improve its predictive accuracy (Alloun et al. 2023).

The neural network architecture designed for predicting the release of aromatic products is provided in **Fig. S17a**. Various network configurations were tested by changing the number of hidden neurons from 1 to 15 to choose the best architecture with the highest overall regression and the lowest Mean Square Error. The optimized neural network model was attained with 7 hidden neurons, resulting in a mean squared error of 0.0021798 after training at epoch 11. Additionally, the model attained an overall correlation coefficient of 0.98 (**Fig. S17b, Fig. S18**).

After analyzing the various factors that impact the response variable and evaluating the model's adequacy, an optimization process was performed using a Genetic Algorithm. The results illustrated in **Fig. S19a** demonstrate the GA's efficiency in exploring the search space and progressively improving solutions over nearly 90 generations. This process led to the convergence of the fitness value, ultimately resulting in the GA approaching the maximum predicted normalized value of 0.90, the equivalent of 1286 μM. This value represents the highest level expected for the aromatic products' release. Achieving this optimal level requires optimal conditions, which are detailed in **Fig. S19b.**

The distribution of observed and predicted concentrations of released products, the performance of the RSM and RSM-ANN models is shown in **Fig. S20**. The predicted values

from the RSM-ANN hybrid model are more closely aligned with the experimental data than those from the standalone RSM model. The coefficient of determination (R^2) for the RSM model was found to be 0.75, while the RSM-ANN hybrid model achieved a higher R^2 value of 0.84, demonstrating improved prediction accuracy

SUPPLEMENTARY NOTES:

Supplementary Note 1: Primers. DNA. and Protein Sequence Information for BhEstB *B. halotolerans* HGR5 strain.

>BhEstB_For

gcgGCTAGCATGTCTGAATCAATGG

>BhEstB_Rev

gcgAAGCTTTGAGTTTAATAGTTTTTTCC

>BhEstB-His6_DNA

ATGTCTGAATCAATGGTCAAAACACAATACGGCACAGTGAAAGGCATTTCAAAAAACGGTGTCC
ACATATGGAAAGGCATCCCCTACGCAAAGCCGCCCGTCGGACAATTGCGCTTTAAAGCACCTG
AGCCGCCGGCGGCCTGGGAAGGCGTACTGGACGCAACTGCCTACGGCCCGGTTTGCCCGCA
GCCGCCTGATTTACTGTCATATTCATACCCTGAACTGCCCCGCCAGTCTGAGGATTGCCTGTAT
GTAAATGTATTTGCGCCTGACACTCCTGGCAAAAATCGTCCTGTCATGGTGTGGATTCACGGC
GGCGCATTTTATCTCGGCGCGGGCAGTGAGCCTTTATACGACGGCTCAAACCTGGCGGCTCT
GGGGGATGTCATTGTCGTCACACTGAACTATCGGCTGGGGCCATTCGGCTTTTTACATTTGTCT
TCCATTGATGAAGCGTATTCCGACAACCTTGGACTGCTTGACCAAACCGCGGCTCTGAAATGG
GTCAGGGACAATATCTCTGCGTTTGGCGGCGATCCGGGAAATGTAACGGTGTTTGGAGAATCA
GCAGGCGGTATGAGCATTGCCGCACTGCTTGCCATGCCTGCTGCAAAAGGCCTGTTCCAGAAA
GCAATCTTGGAAAGCGGATCTTCCCGAACCATGACGGAAGAAAAAGCGGCCAGCACCGCGGA
TGCTTTTTTACGAGTCCTTGGAATTGAACGTCACCAATTGGACAGGCTGCATACTGTGTCTGAG
GAAGATTTGCTTAAAGCCGCCGACCAGCTGCGAAAAACCGAAAATGAAAATATCTTTCAGCTGT
TCTTCCAGCCTGCACTTGATCCGAAAACACTGCCTGCTGAGCCGGAGAAAGCCATTGCTCAAG
GGGCCGCTGACGGCATTCCGCTGTTAGTCGGAACAAATCGTGATGAAGGGTATTTATTTTTTAC
GCCGGATTCAGAAGTTCACTCTCAGGAAACGATAGATGAAGCGCTTGAGTATCTATTGGGGCA
GCCGCTGGCAAAGAAAGCGGCCGATCTGTATCCGCGCTCTCTCGAAAGCCAAATTCATATGAT
GACTGATTTGCTGTTTTGGCGCCCGGCCGTCGCCTGCGCCTCCGCCCAGTCCCGTTACGCAC
CTGTCTGGATGTACCGATTCGACTGGCATCCGGACAAGCCGCCGTACAACAAAGCGTTTCACG
CACTGGAACTGCCGTTTGTGTTCGGCAATCTAAACGGACTGAAACGAATGGTACAAGCAGACA
TTACGGATGAGGTCAAACAGCTCTCACACACGATACAATCAGCATGGCTTGCCTTCGCAAAAAC
AGGAAATCCAAGCTGCGAGGATGTACAATGGCCGGTTTATACTGAAGACAAACGAGAAACCTT
GATTTTGAATTGCGAACTTTCTATTGAGCATGATCCTGATGGTGAGAAGCGGAAAAAACTATTA
AACTCAAAGCTTGCGGCCGCACTCGAGCACCACCACCACCACCAC

>**BhEstB-His6_Protein**

MSESMVKTQYGTVKGISKNGVHIWKGIPYAKPPVGQLRFKAPEPPAAWEGVLDATAYGPVCPQPP
DLLSYSYPELPRQSEDCLYVNVFAPDTPGKNRPVMVWIHGGAFYLGAGSEPLYDGSNLAALGDVIV
VTLNYRLGPFGFLHLSSIDEAYSDNLGLLDQTAALKWVRDNISAFGGDPGNVTVFGESAGGMSIAA
LLAMPAAKGLFQKAILESGSSRTMTEEKAASTADAFLRVLGIERHQLDRLHTVSEEDLLKAADQLRK
TENENIFQLFFQPALDPKTLPAEPEKAIAQGAADGIPLLVGTNRDEGYLFFTPDSEVHSQETIDEALE
YLLGQPLAKKAADLYPRSLESQIHMMTDLLFWRPAVACASAQSRYAPVWMYRFDWHPDKPPYNK
AFHALELPFVFGNLNGLKRMVQADITDEVKQLSHTIQSAWLAFAKTGNPSCEDVQWPVYTEDKRET
LILNCELSIEHDPDGEKRKKLLNSKLAAALEHHHHHH

Supplementary Note 2: ConSurf-Colored Sequence of BhEstB. It provides information on the conservation level of each amino acid. as well as whether each residue is exposed or buried and its contribution to the function or structure of the protein. The 3D visualization of this analysis. using the same color scale. is shown in panel **(c)** of Supplementary **Fig.**04**.**

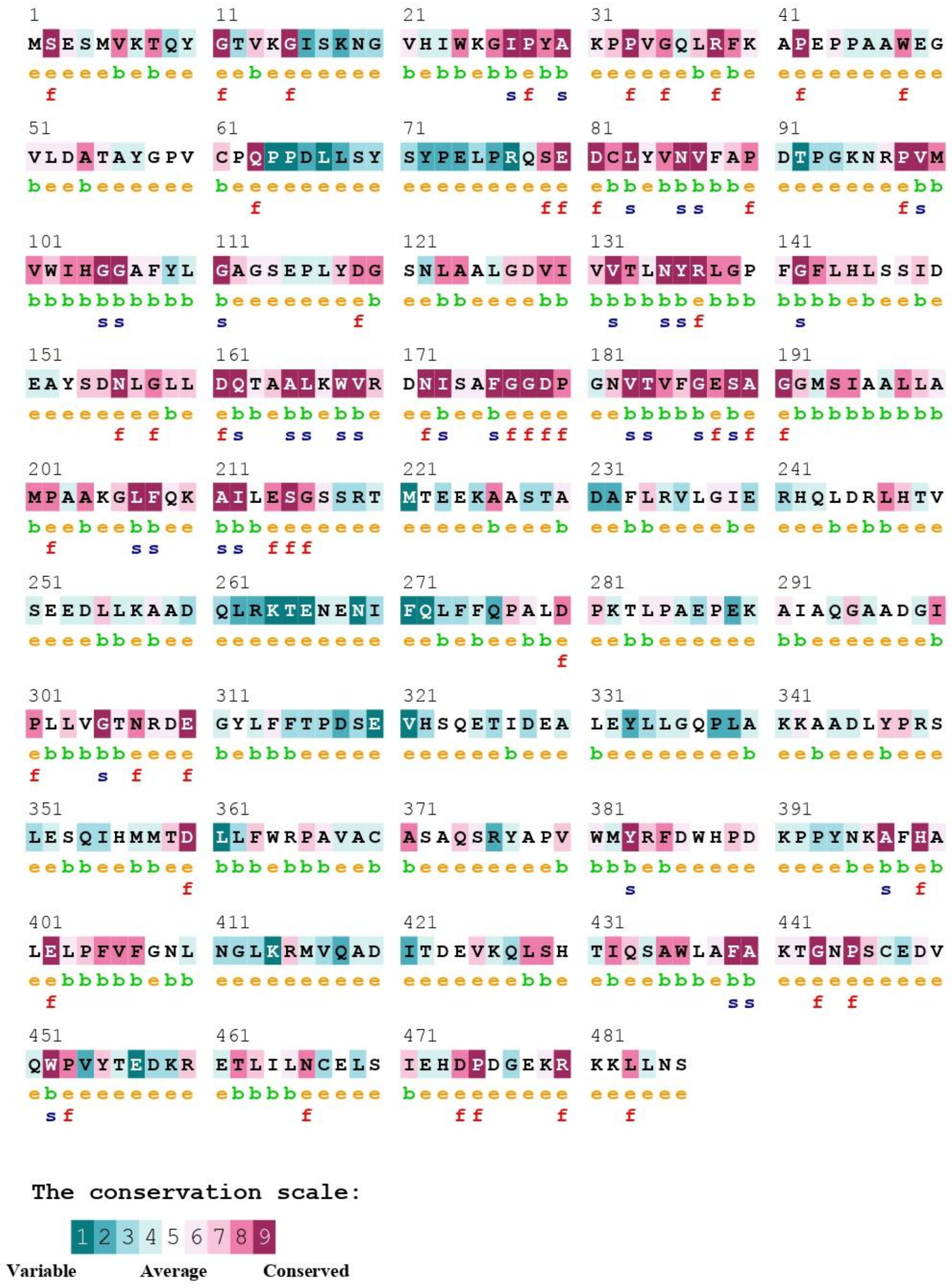

e - An exposed residue according to the neural network algorithm.

b - A buried residue according to the neural network algorithm.

f - A predicted functional residue (highly conserved and exposed).

s - A predicted structural residue (highly conserved and buried)

SUPPLEMENTARY TABLES

Supplementary Table 1: sum of aromatic products (TPA. MHET and BHET) released from a 24-hour PET powder degradation by different enzymes at their respective optimal temperatures (40 °C for IsPETase. 50 °C for LCC and 60 °C for BhEstB).

Enzyme	Phylum	Released aromatic products (μM /$μM_{enzyme}$ /day)
BhEstB	Bacillota	229.83 ± 73.39
LCC	Actinomycota	1097.49 ± 192.19
IsPETase	Pseudomonadota	758.411 ± 22.22

Supplementary Table 2: CCD experimental runs with variable combinations and measured responses for total aromatic products releases from PET degradation by BhEstB, all responses were normalized between 0 and 1.

#	T (X1)	pH (X2)	[BhEstB] (X3)	Relased aromatic products (μM)	Normalised response
1	-1	-1	-1	368.22	0.192
2	1	-1	-1	127.91	0.006
3	-1	1	-1	890.32	0.595
4	1	1	-1	233.16	0.088
5	-1	-1	1	710.87	0.456
6	1	-1	1	155.74	0.028
7	-1	1	1	996.67	0.677
8	1	1	1	277.19	0.122
9	-2	0	0	211.98	0.071
10	2	0	0	264.10	0.112
11	0	-2	0	378.05	0.199
12	0	2	0	794.28	0.521
13	0	0	-2	193.96	0.057
14	0	0	2	921.22	0.619
15	0	0	0	1020.12	0.695
16	0	0	0	963.22	0.651
17	0	0	0	691.30	0.441
18	0	0	0	1026.97	0.700
19	-1	-1	-1	499.46	0.293
20	1	-1	-1	119.56	0.000
21	-1	1	-1	624.00	0.389
22	1	1	-1	190.01	0.054
23	-1	-1	1	1024.42	0.698
24	1	-1	1	206.70	0.067
25	-1	1	1	1197.26	0.832
26	1	1	1	729.87	0.471
27	-2	0	0	350.56	0.178
28	2	0	0	244.00	0.096
29	0	-2	0	195.86	0.059

30	0	2	0	807.49	0.531
31	0	0	-2	216.00	0.074
32	0	0	2	1415.44	1.000
33	0	0	0	910.38	0.610
34	0	0	0	768.14	0.500
35	0	0	0	941.10	0.634
36	0	0	0	685.48	0.437
37	-1	-1	-1	263.41	0.111
38	1	-1	-1	181.32	0.048
39	-1	1	-1	483.12	0.281
40	1	1	-1	366.17	0.190
41	-1	-1	1	508.81	0.300
42	1	-1	1	206.70	0.067
43	-1	1	1	1160.99	0.804
44	1	1	1	386.97	0.206
45	-2	0	0	371.80	0.195
46	2	0	0	226.99	0.083
47	0	-2	0	218.22	0.076
48	0	2	0	579.18	0.355
49	0	0	-2	238.05	0.091
50	0	0	2	1294.45	0.907
51	0	0	0	671.65	0.426
52	0	0	0	784.20	0.513
53	0	0	0	1134.09	0.783
54	0	0	0	982.78	0.666

Supplementary Table 3: ANOVA summary of the RSM model used for the optimization of PET degradation.

Source		DF	Adj SS	Adj MS	F-Value	P-Value
**Model**		9	5236697	581855	15.02	0.000
	**Linear**	3	3227713	1075904	27.77	0.000
	**Temperature**	1	736247	736247	19	0.000
	**pH**	1	735132	735132	18.97	0.000
	**[enzyme]**	1	1756334	1756334	45.33	0.000
	**Square**	3	1837883	612628	15.81	0.000
	**Temperature*Temperature**	1	1618350	1618350	41.77	0.000
	**pH*pH**	1	693360	693360	17.89	0.000
	**[enzyme]*[enzyme]**	1	153991	153991	3.97	0.052
	**2-Way Interaction**	3	171101	57034	1.47	0.235
	**Temperature*pH**	1	26119	26119	0.67	0.416
	**Temperature*[enzyme]**	1	124050	124050	3.2	0.08
	**pH*[enzyme]**	1	20932	20932	0.54	0.466
**Error**		44	1704843	38746		
	**Lack-of-Fit**	5	832618	166524	7.45	0.000
	**Pure Error**	39	872225	22365		
**Total**		53				

Supplementary Table 4: Optimal conditions predicted by GA for the highest PET degradation.

Variable	Temperature (°C)	pH	[BhEstB] (μM)
**Optimal condition**	60.58	8.515	3

SUPPLEMENTARY FIGURES:

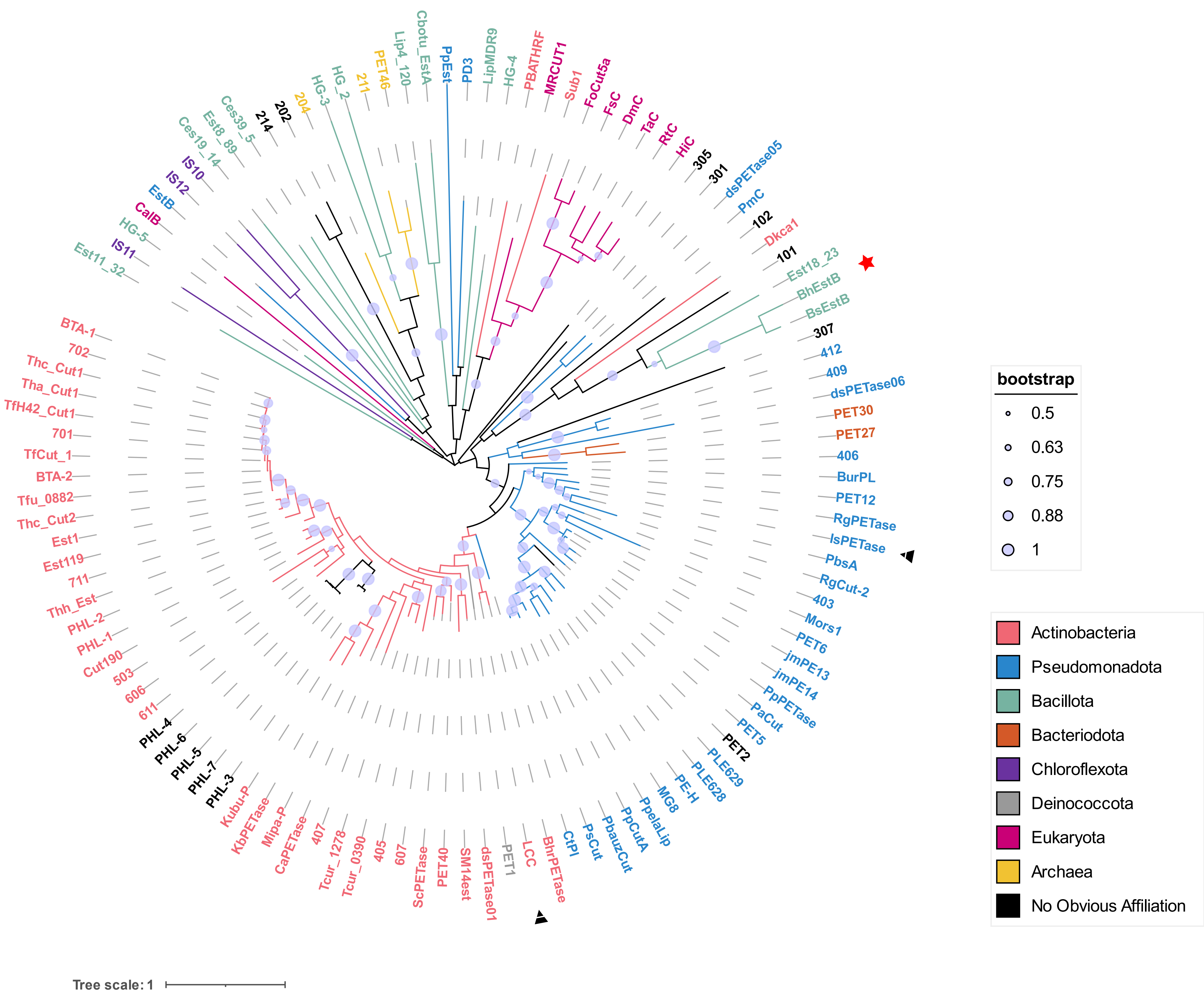

Supplementary Fig. 1: A phylogenetic tree of PET hydrolases retrieved from PAZy on October 2025, primarily consisting of Actinobacteria (blush pink) and Pseudomonadota (blue). The remaining enzymes, which are more distantly related, represent a diverse range of bacterial, archaeal, and eukaryotic taxa, shown in various colors. BhEstB (marked with a star) cluster together with BsEstB.

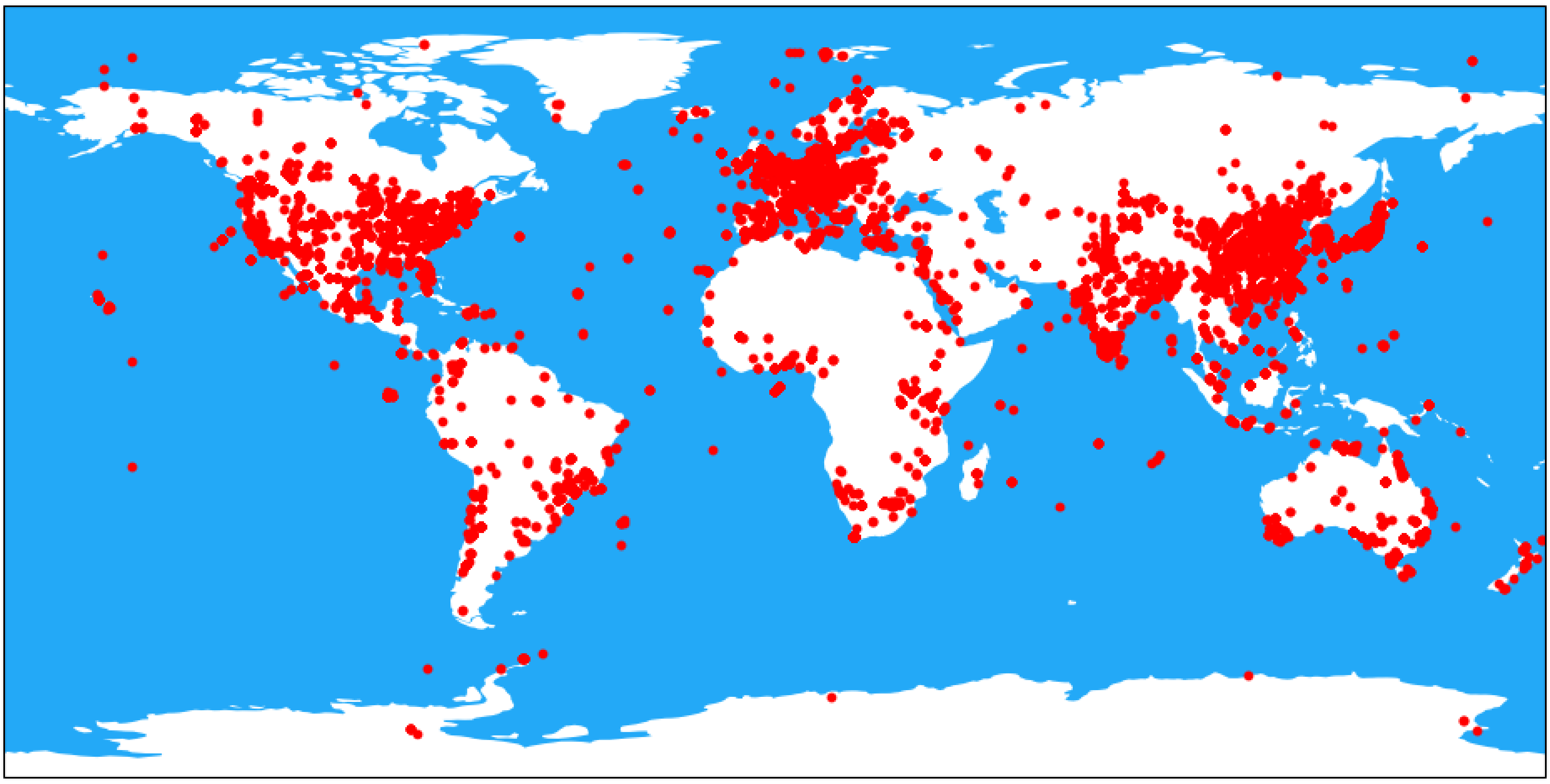

Supplementary Fig. 2: Global distribution of the *Bacillus subtilis* group displayed on a world map. Red dots represent locations where sequences belonging to this clade were identified in publicly available (meta)genomic datasets. Data was downloaded on 04.03.2026 from MicrobeAtlas Version 1.0, and the map was created using cartopy on Python.

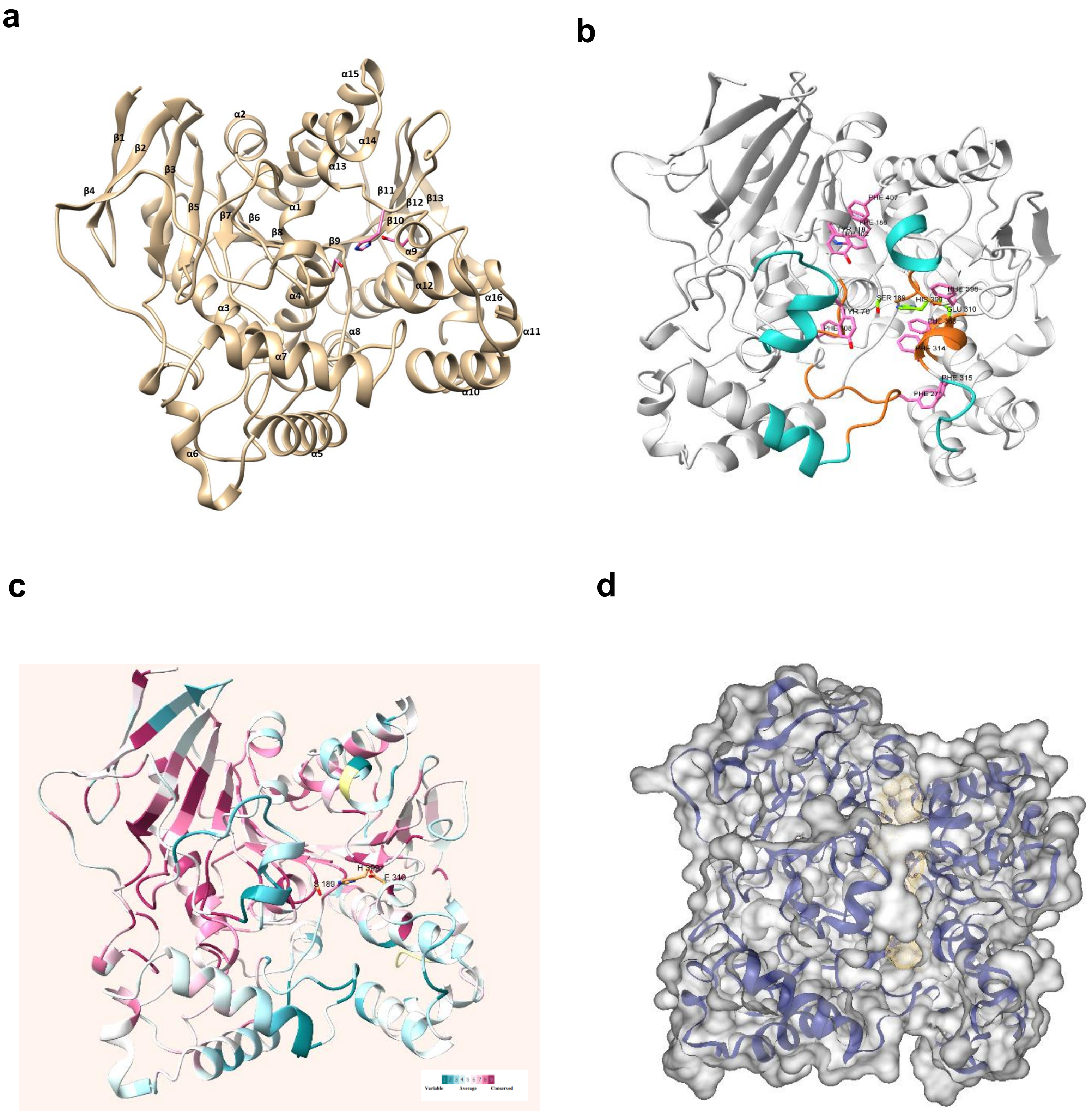

Supplementary Fig. 3: BhEstB 3D Structure Visualization. (a) 3D structure of BhEstB **(b)** The 3D structure of BhEstB with the active site residues highlighted in orange and the entrance to the active site in cyan. These predictions are based on work from (1999 paper), considering the high structural similarity between the two proteins. Additionally, atoms of the catalytic triad and aromatic residues involved in substrate binding are visualized. **(c)** Conservation analysis using ConSurf server, comparing BhEstB with homologous sequences, shows that all 2D structural elements shared with other PETases are mostly conserved, including residues involved in MHET/BHET/PET binding and degradation. In contrast, unique structural features of BhEstB exhibit low to high variability, suggesting they are not essential for enzymatic activity. Data supporting this analysis are provided in **Supplementary Note 02**. **(d)** Binding pocket predictions were made using DoGSiteScorer from the Protein+ server. The surface area of the active site pocket is shown in yellow. Supporting data for this analysis are available in the **Figure** data Excel file.

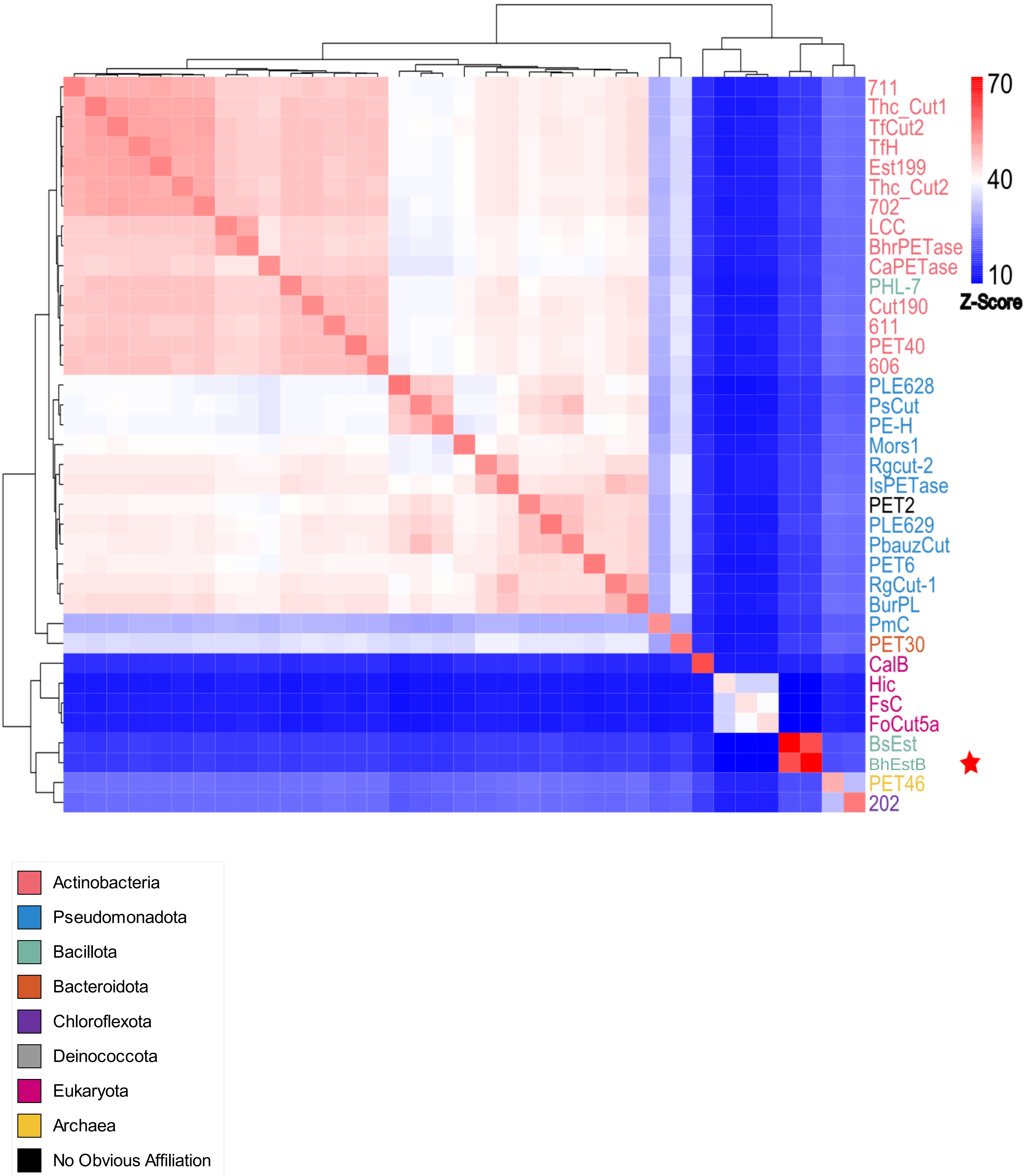

Supplementary Fig.04: A heatmap of structural similarities between various PET esterases. The largest cluster is predominantly composed of bacterial enzymes, with a noticeable subcluster of Actinobacteria (blush pink), including the LCC. Notably, BhEstB (marked with a star) and BsEst from *B. subtilis* are very similar, and are structurally distinct from bacterial PETases, exhibiting low similarity scores similar to enzymes from Eukarya and Archaea. Relevant supporting data are available in the figure data Excel file.

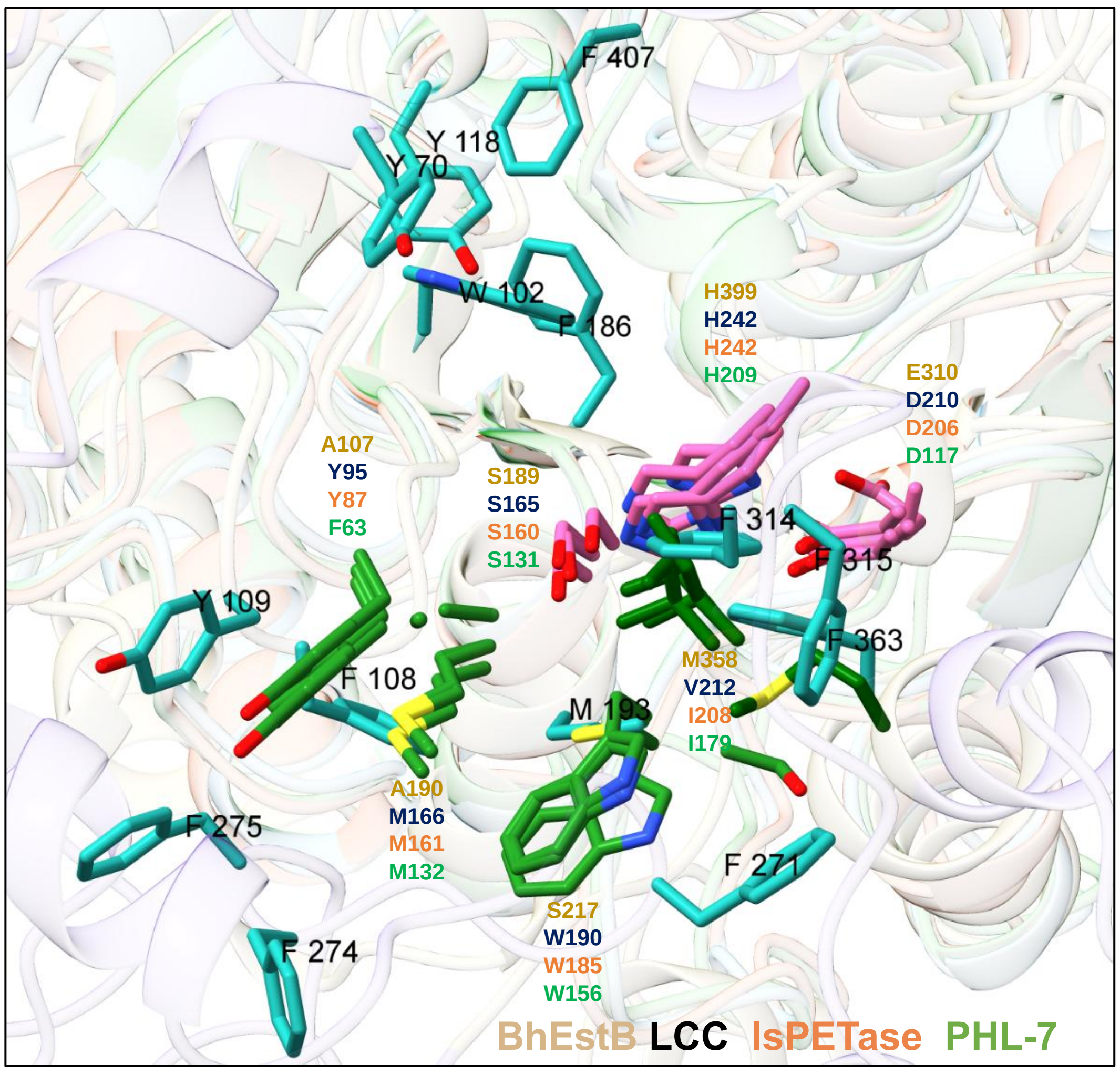

Supplementary Fig. 5: A close-up view of the active sites in four PET esterases shows a conserved catalytic triad. BhEstB, however, contains a glutamate (Glu) residue in place of aspartate (Asp), lacks the amino acids required for PET binding, and instead has several aromatic amino acids near the active site.

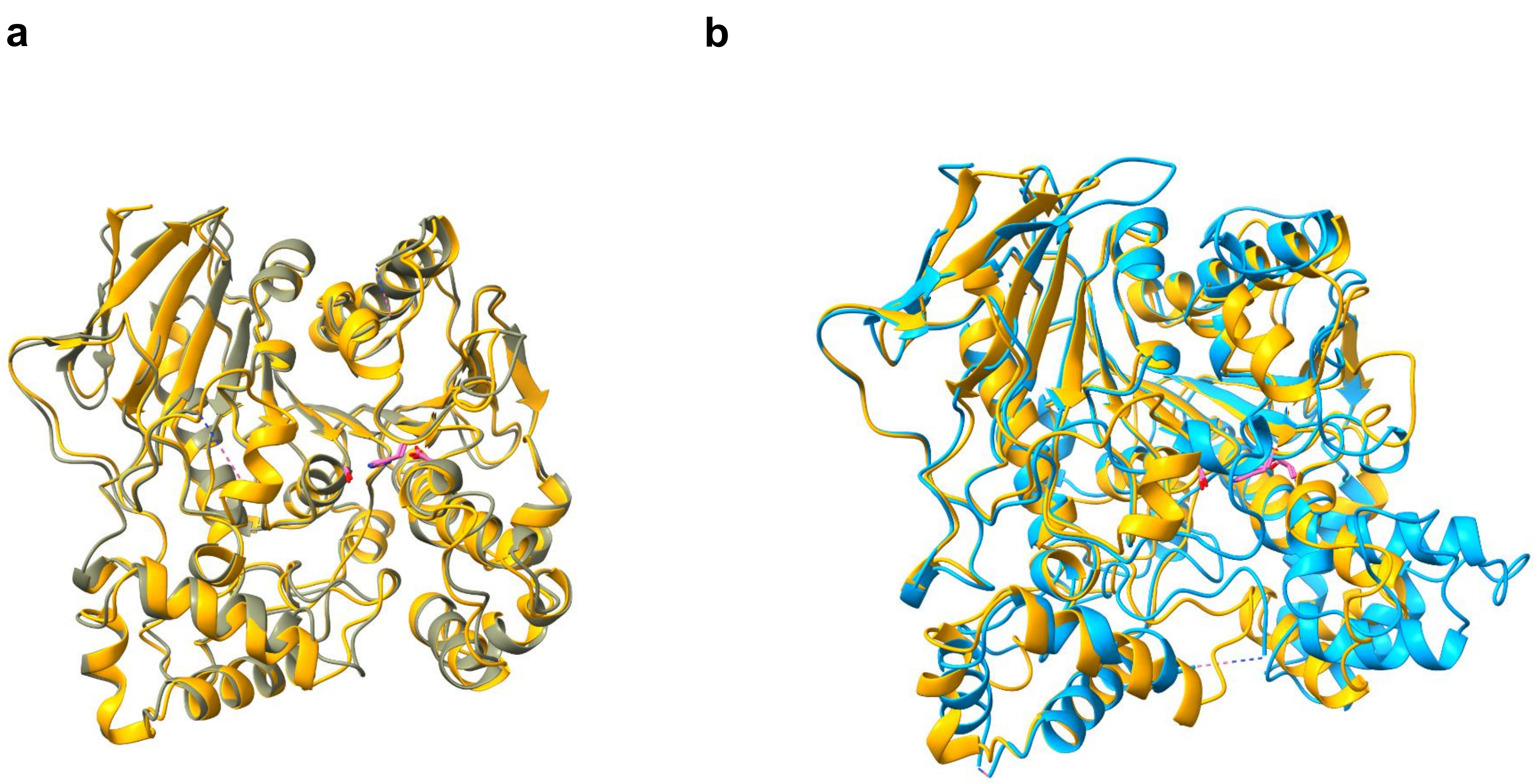

Supplementary Fig. 6: BhEstB (amber) 3D structural comparisons. Comparison with BsEstB from B. subtilis (olive gray) demonstrates that both enzymes exhibit high structural similarity, with only minor differences. This similarity is consistent with their high sequence identity **(a)**. Alignment of BhEstB with human AChE (cyan-blue) indicates structural similarity, particularly in the catalytic triad, where the same amino acids are conserved **(b)**.

a

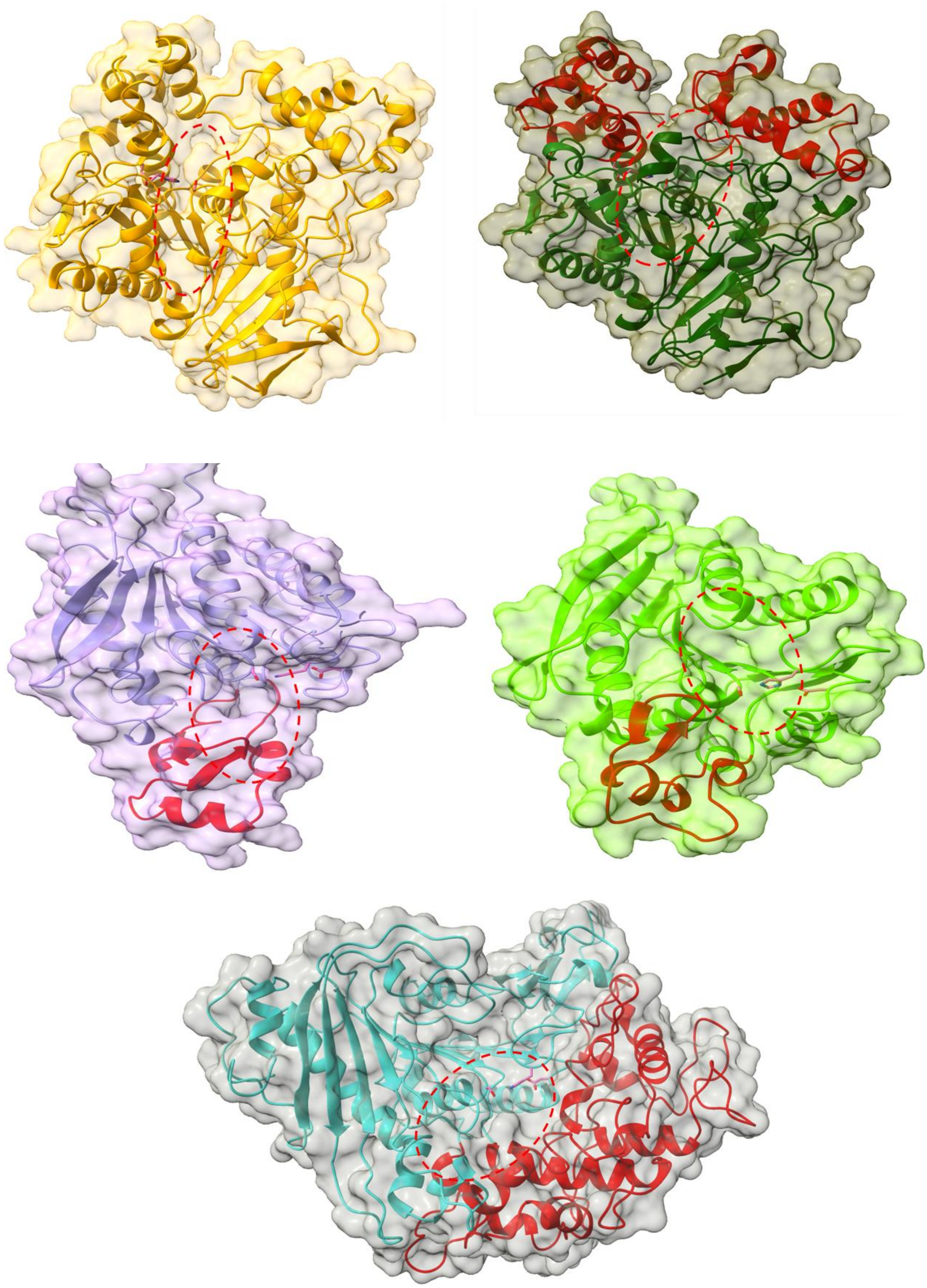

Supplementary Fig. 7: Predicted substrate-binding grooves of BhEstB **(a)**, TfCa **(b)**, PET46 **(c)**, MHT077 **(d)**, and IsMHETase **(e)**. Catalytic triad residues are shown as hot pink sticks. The putative substrate-binding grooves are indicated by red circles based on the corresponding pocket predictions from DoGSiteScorer via the Protein+ server.

a

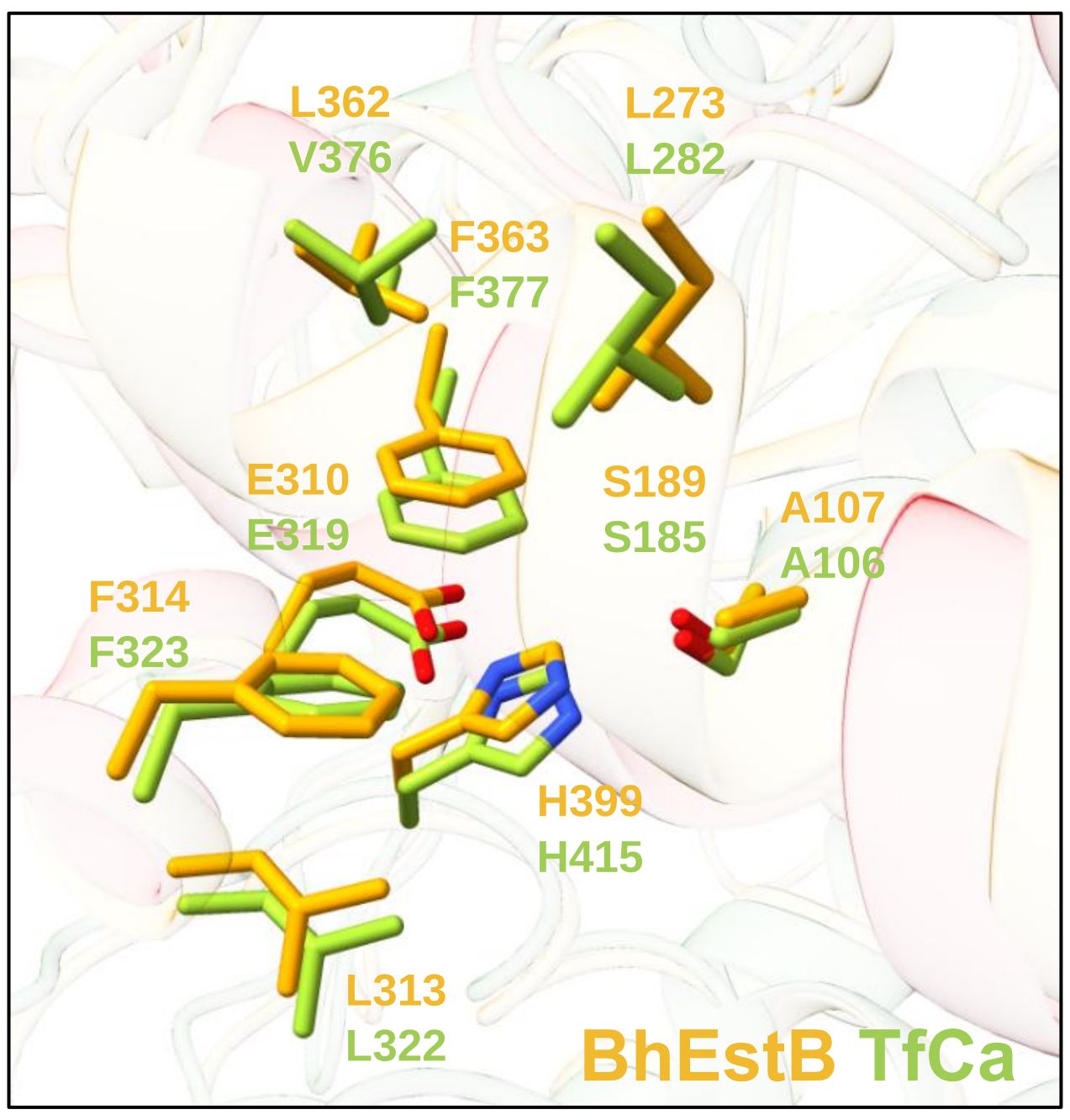

b

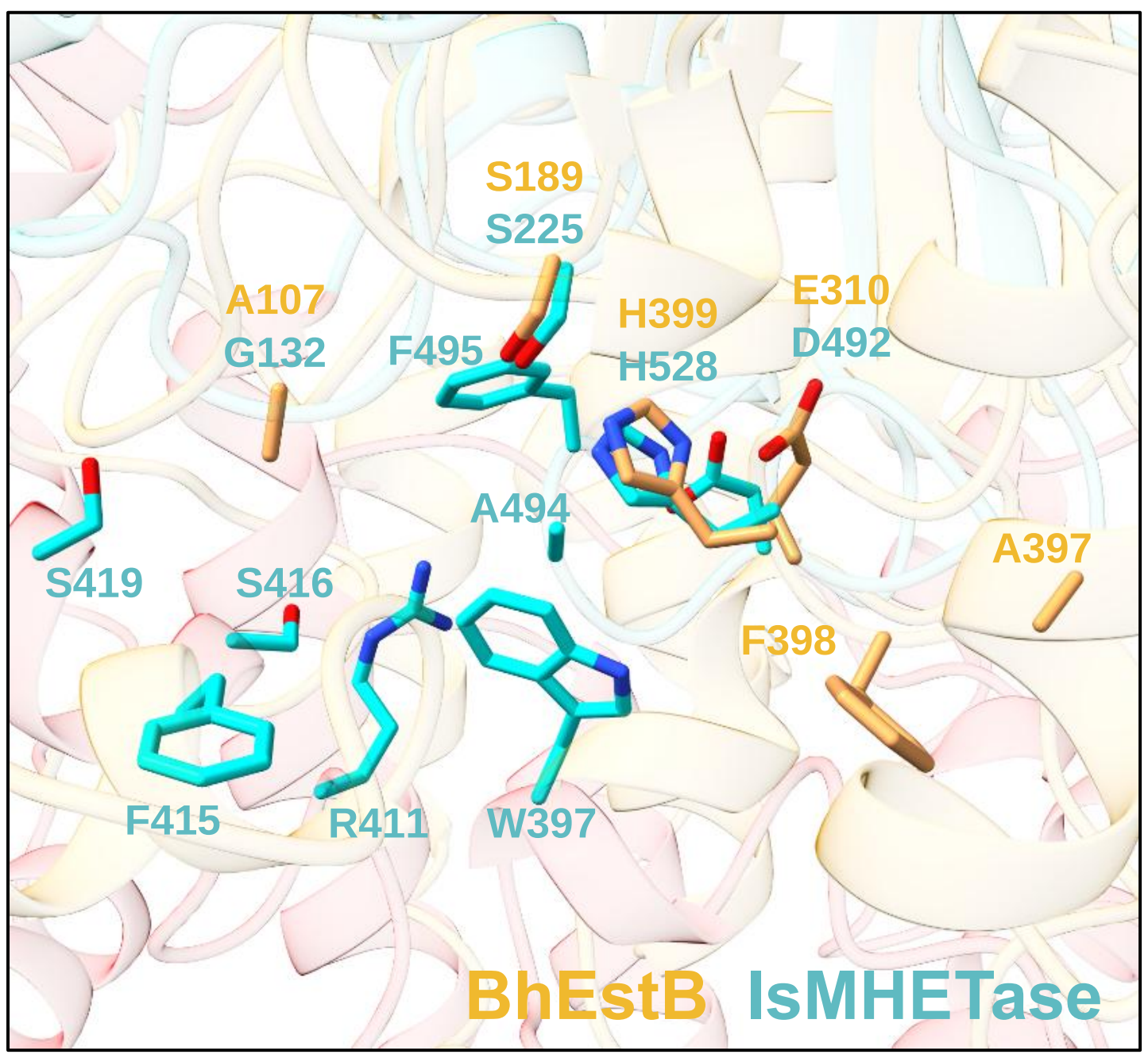

Supplementary Fig. 8: (a) A close-up view of the active sites in aligned BhEstB and TfCa shows a similar composition of the catalytic triad. Additionally, all amino acids known to contribute to MHET binding in TfCa are present in BhEstB (von Haugwitz et al. 2022). **(b)** In contrast, a close-up of the active sites in aligned BhEstB and IsMHETase reveals a complete divergence in MHET binding amino acids. While A494 and F495 have exact equivalents in BhEstB (A397 and F398), they are located on the opposite side of the active site. G132 is replaced by A107 in BhEstB. Furthermore, all residues from the IsMHETase lid (W397, R411, F415, S416, and S419) that contribute to MHET binding and activity are absent in BhEstB, which lacks a comparable lid (Magalhães et al. 2021; Liu et al. 2023).

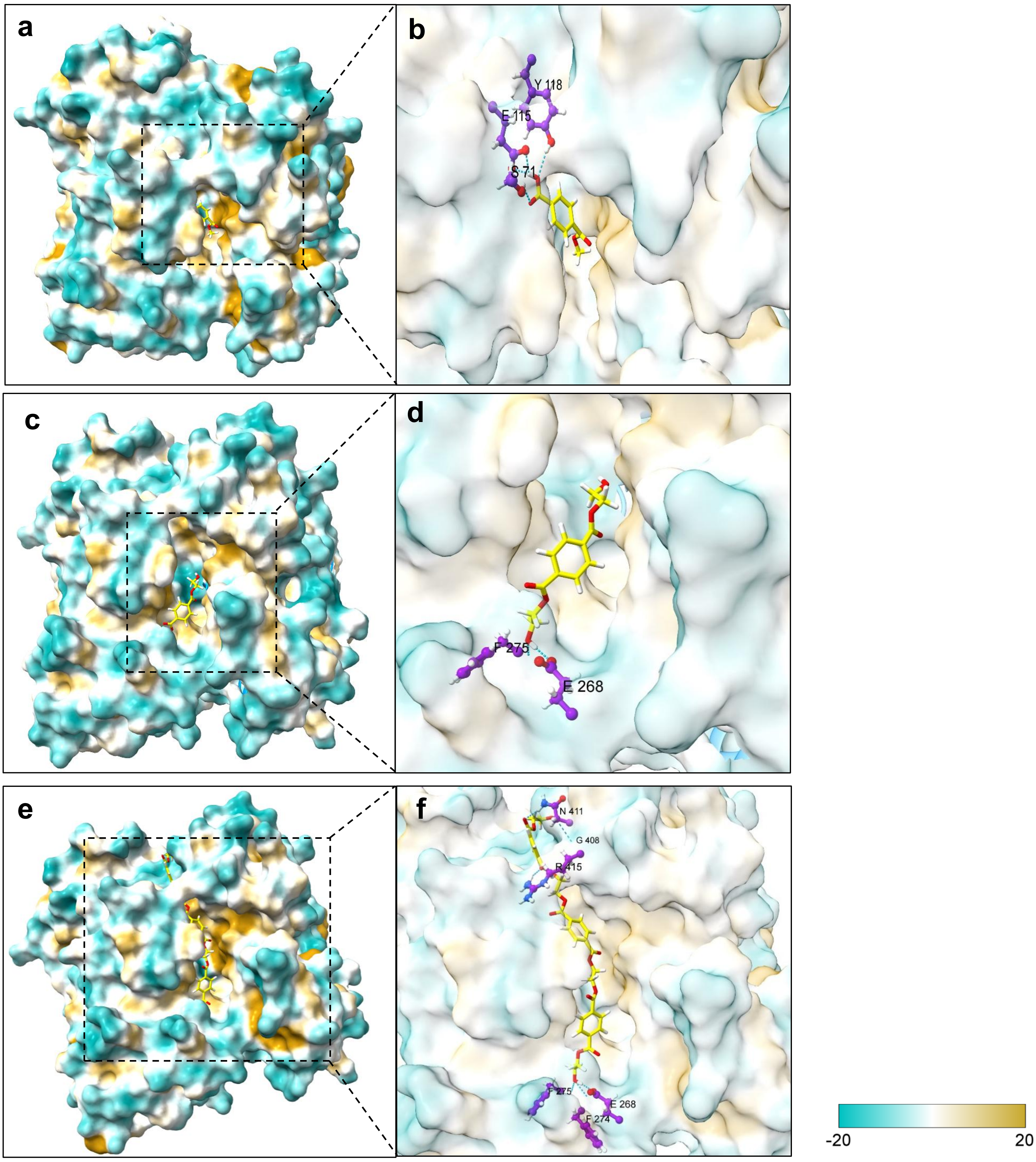

Supplementary Fig. 9: Molecular Docking of PET Building Blocks into the Active Site of BhEstB. **(a)**, **(c)**, and **(e)** show full enzyme structures displayed in hydrophobic mode, with MHET, BHET, and 3PET docked into the active site, respectively. Meanwhile, **(b)**, **(d)**, and **(f)** provide close-up views of the docked substrates, showcasing the amino acids interacting through hydrogen bonding with the substrate. These interactions were predicted using ChimeraX v.1.9.

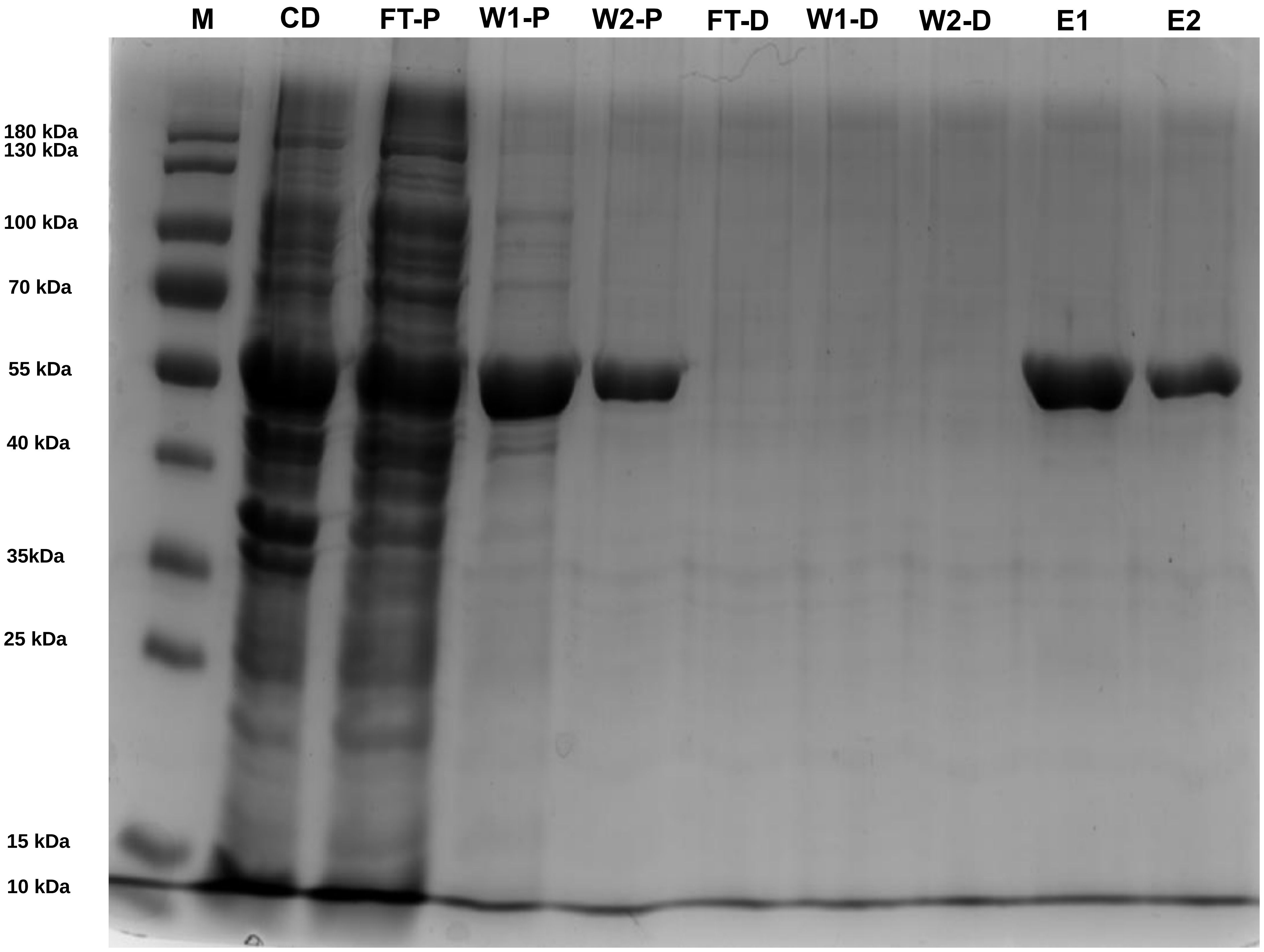

Supplementary Fig. 10: Heterologous expression and purification of BhEstB. SDS-PAGE gel of the enzyme expressed in *E. coli* BL21 (DE3) carrying the pET21a(+) plasmid containing its gene, purified via affinity chromatography, and dialyzed with phosphate buffer. Results were reproduced several times independently. **M :** Marker (PageRuler™ Prestained Protein Ldder); **CD :** Cell Debris pellet; **FT-P :** Flow Through Purification; **W1-P :** Wash1 Purification; **W2-P :** Wash1 Purification; **FT-P :** Flow Through Dialyse; **W1-P :** Wash1 Dialyse; **W2-P :** Wash1 Dialyse; **E1 :** Enzyme diluted 1/10; **E2 :** Enzyme diluted 1/20.

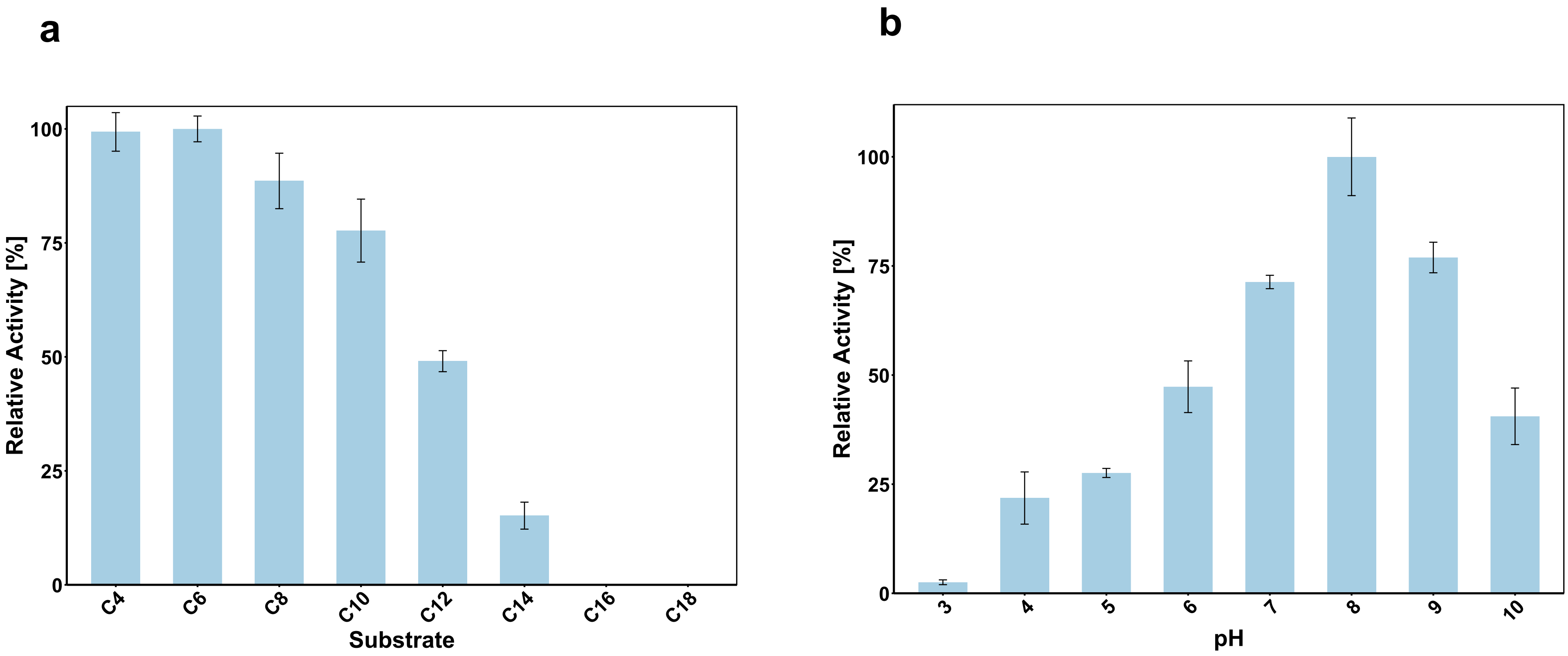

Supplementary Fig 11: Biochimecal characterization of BhEstB. (a). Chain-length substrate preference determined by incubation with *p*NP-ester substrates ranging from C4 to C18, BhEstB showed the highest degradation activity on substrates C4 to C10. **(b).** pNP-ester C10 assay reveals that BhEstB is most active at pH 8.

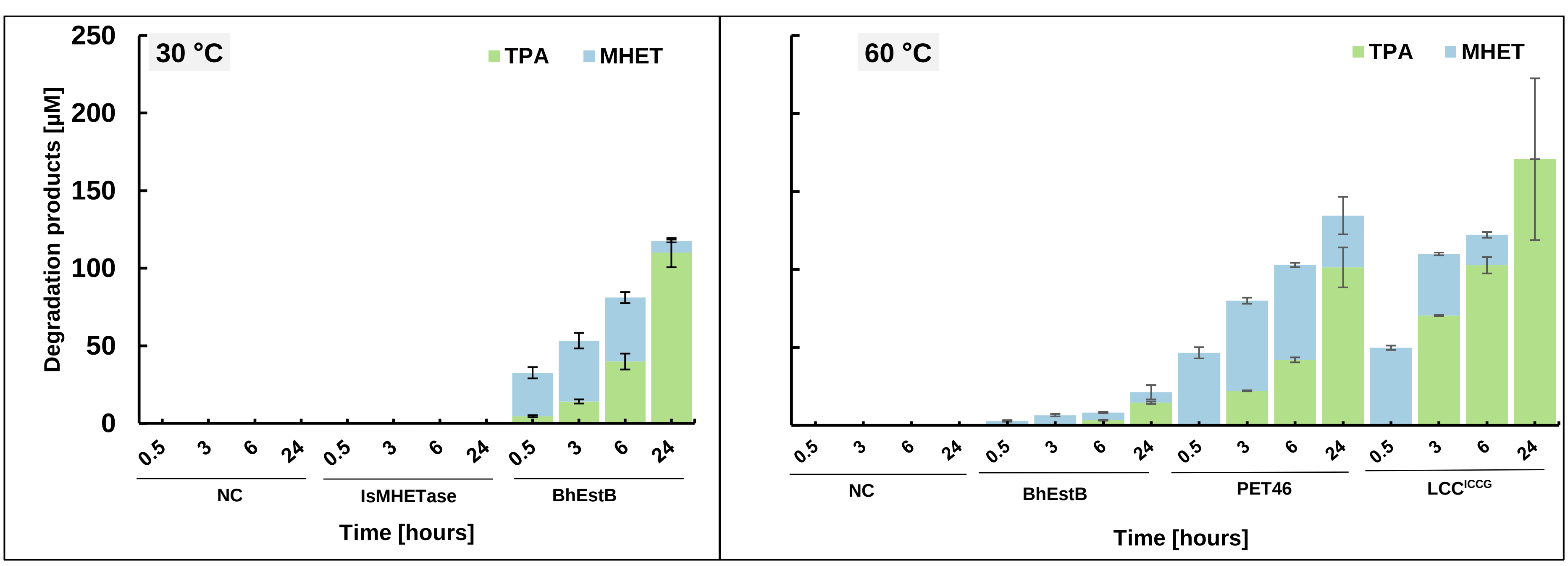

Supplementary Fig. 12: Degradation of 150 µM 3PET over 24 hours by various enzymes under different temperature conditions

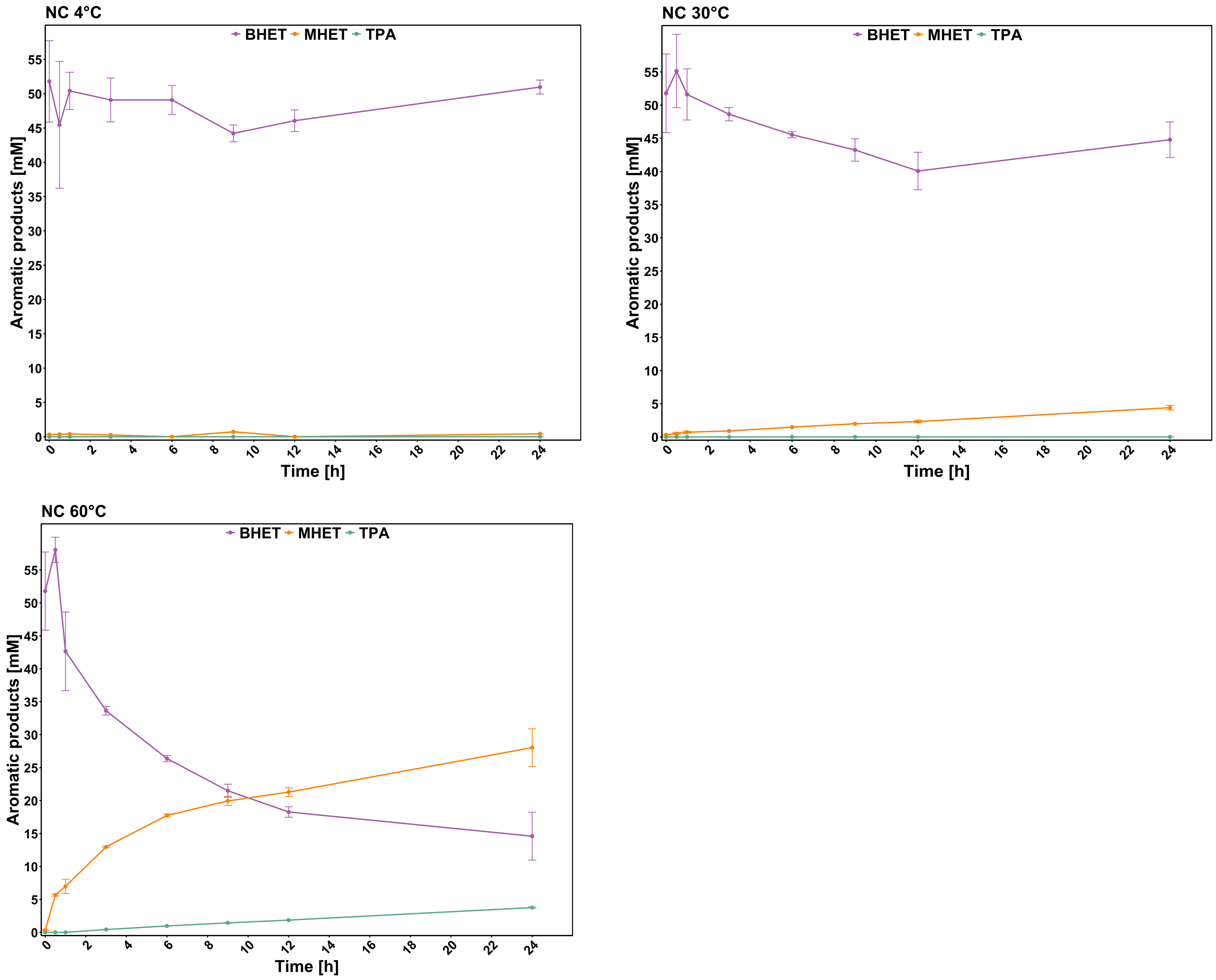

Supplementary Fig. 13: Stability of 50 mM BHET in enzyme-free conditions, across all tested temperatures over 24 hours.

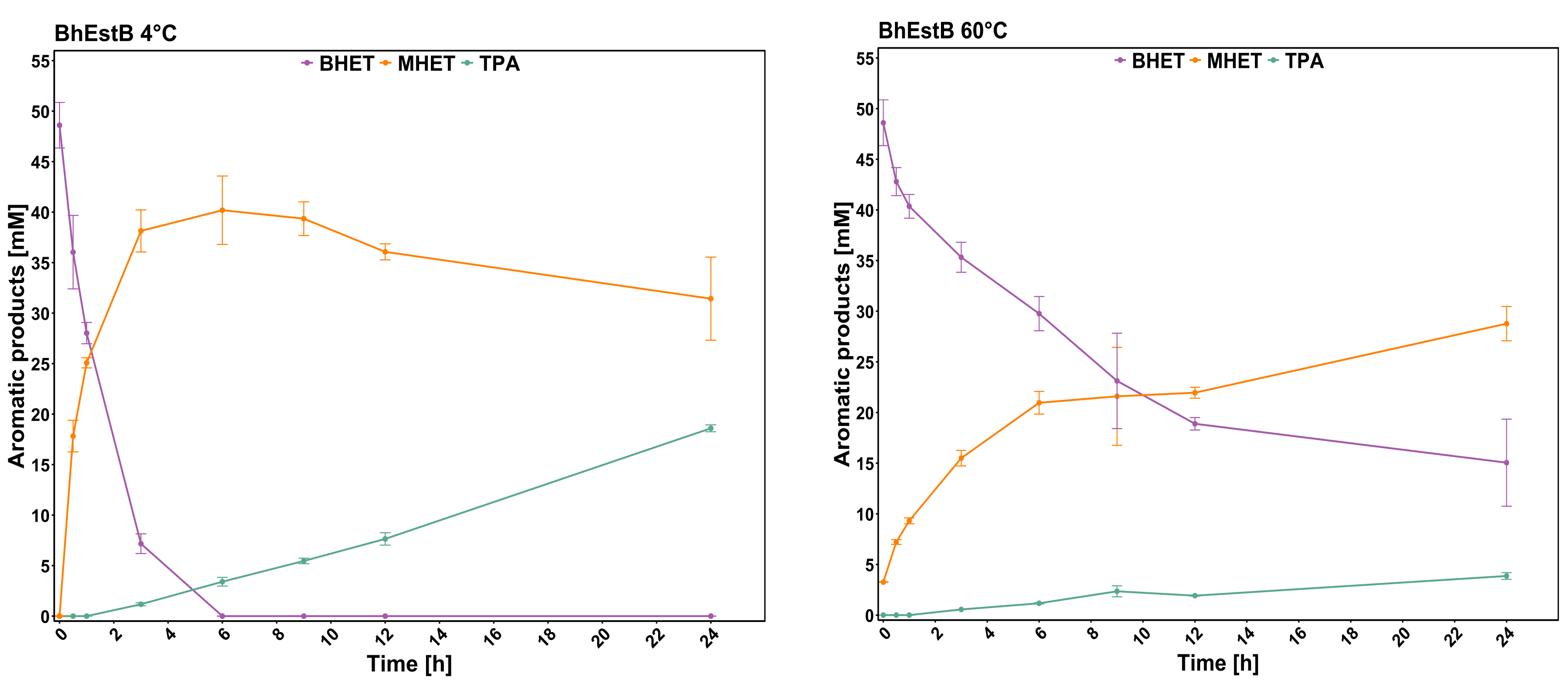

Supplementary Fig. 14: Degradation of 50 mM BHET over 24 hours by BsEstB under different temperature conditions.

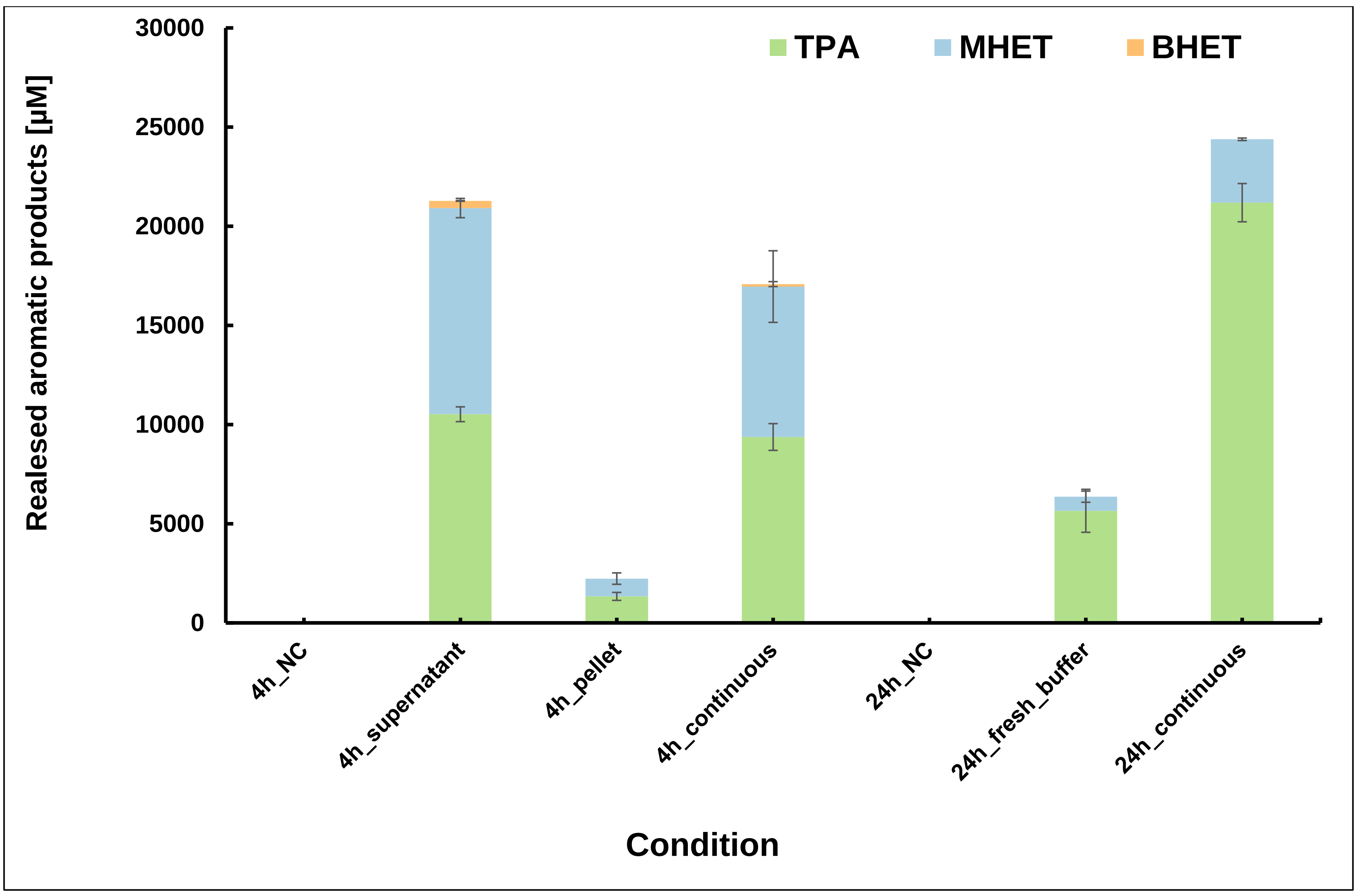

Supplementary Fig. 15: PET powder degradation profiles in enzyme-free conditions or with 3µM of LCC^{ICCG}, assessed under two setups: continuous degradation for 24 hours, and interrupted degradation, where experiments were stopped after 4 hours, the supernatant was replaced with fresh buffer, and incubation continued until 24 hours. Degradation was measured at both 4 and 24 hours for all conditions.

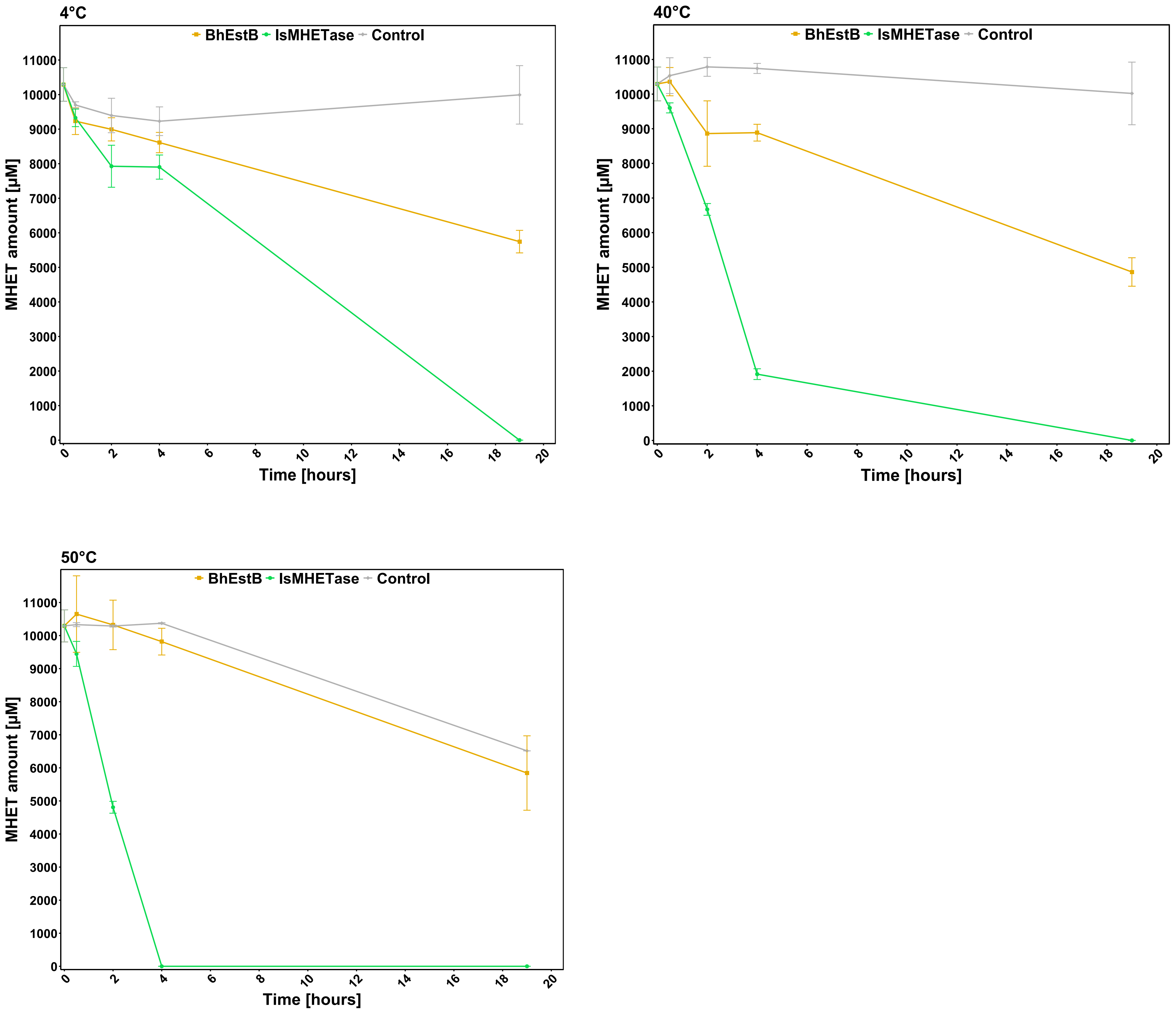

Supplementary Fig. 16: Degradation of 10 mM MHET by various enzymes over 20 hours at several temperatures. A control without any added enzyme was included at each temperature condition.

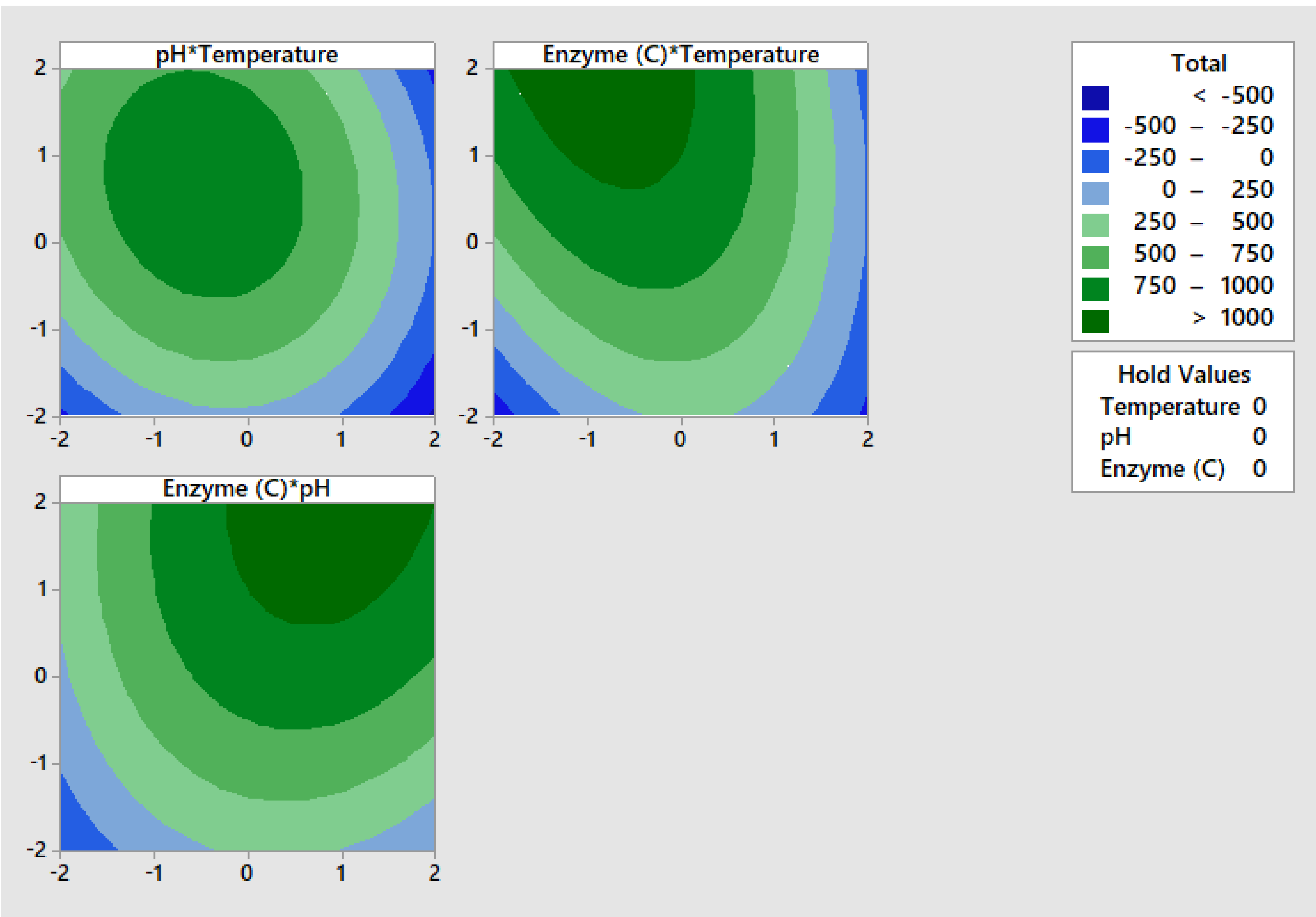

Supplementary Fig. 17: Contour plots of 2-factor interactions influencing PET degradation by BhEstB, indicating the maximum response for each factor combination

a

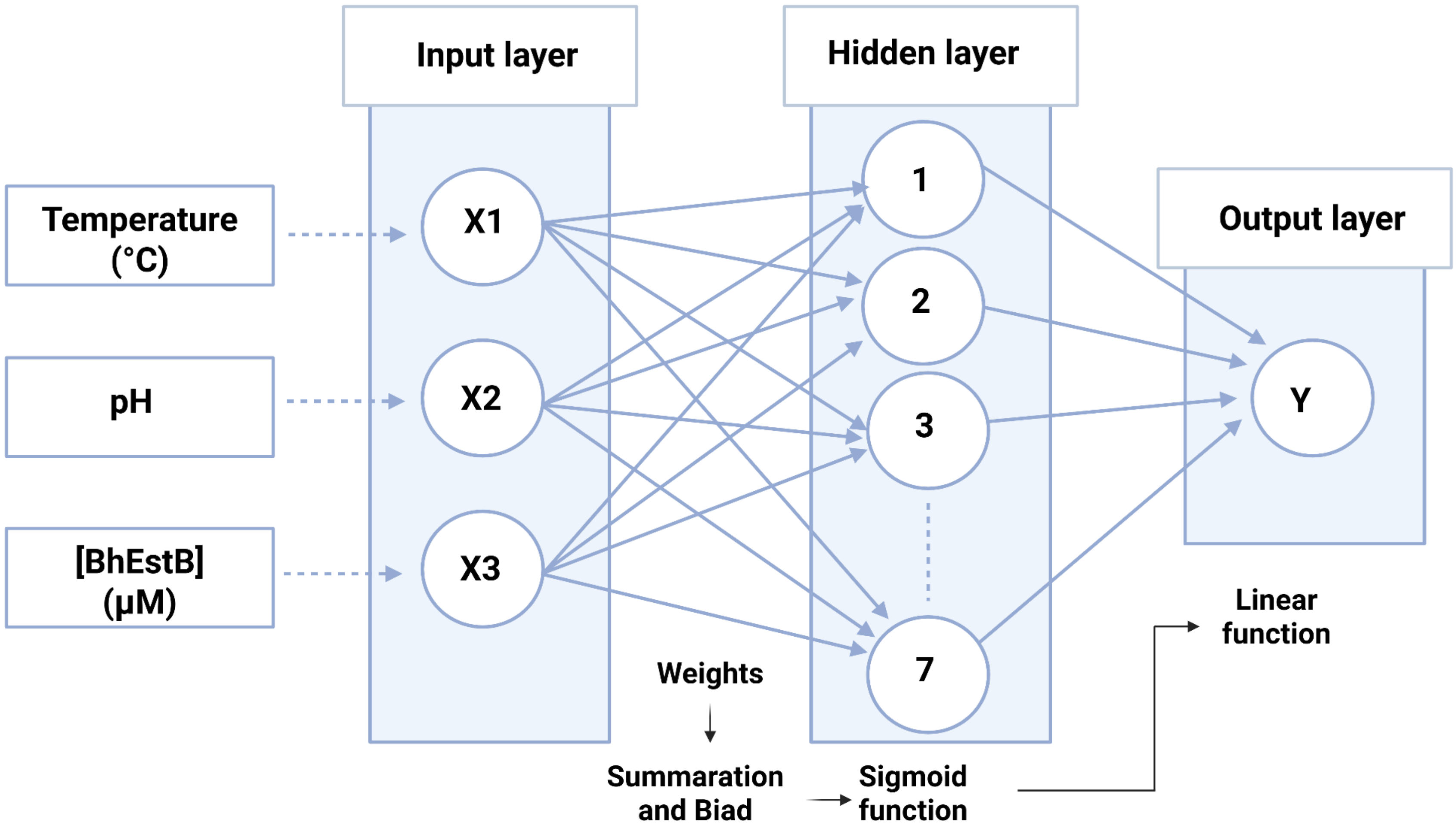

b

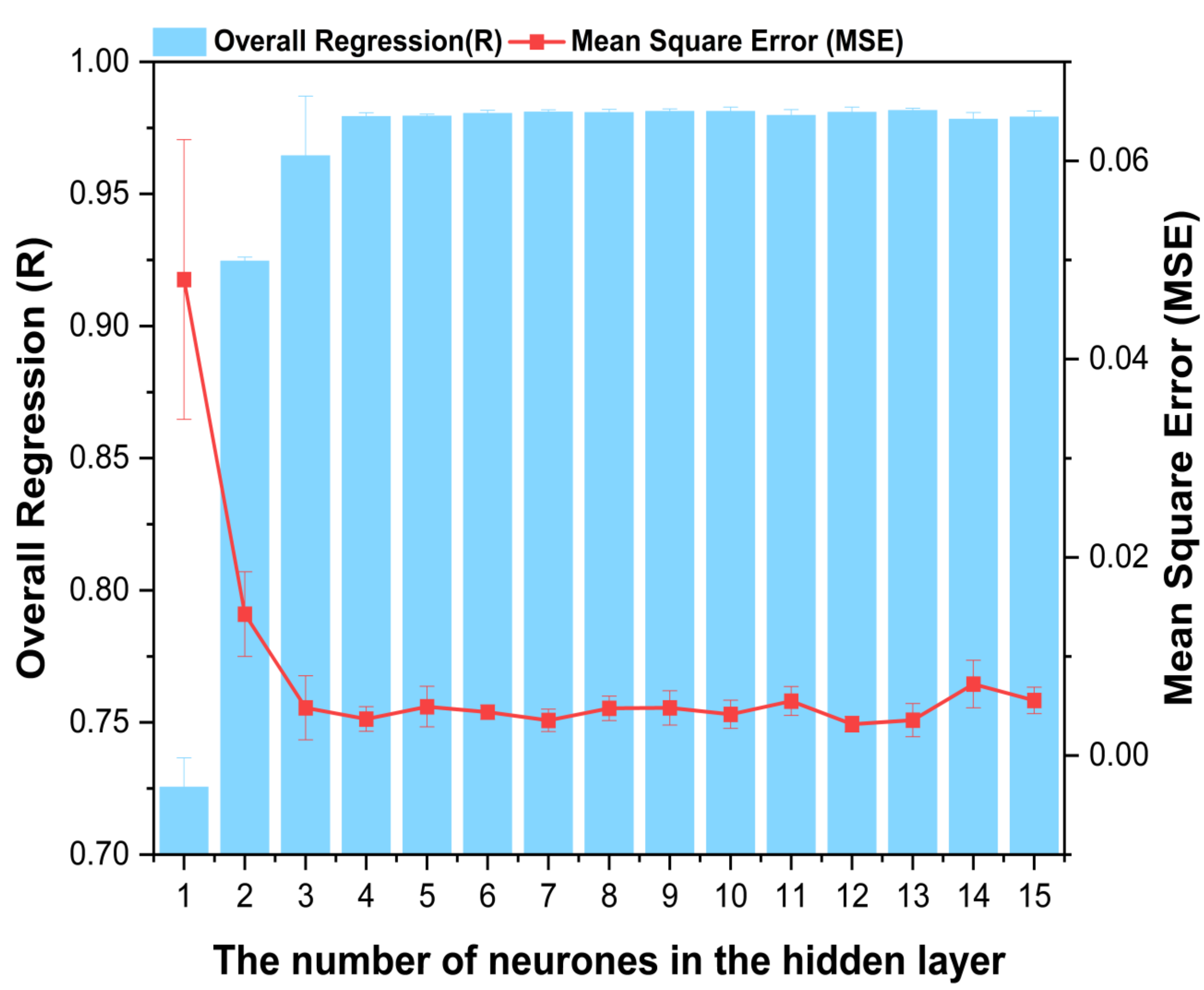

Supplementary Fig. 18: (a) Topology architecture of the Artificial Neural Network (ANN) model. **Fig.**created in Biorender. **(b)** Optimal selection of neuron counts in the hidden layer of the neural network model based on the highest overall regression coefficient (R) and the lowest Mean Square Error (MSE), demonstrating that starting from 4 hidden neurons, the regression coefficient approaches 1 and the error decreases significantly.

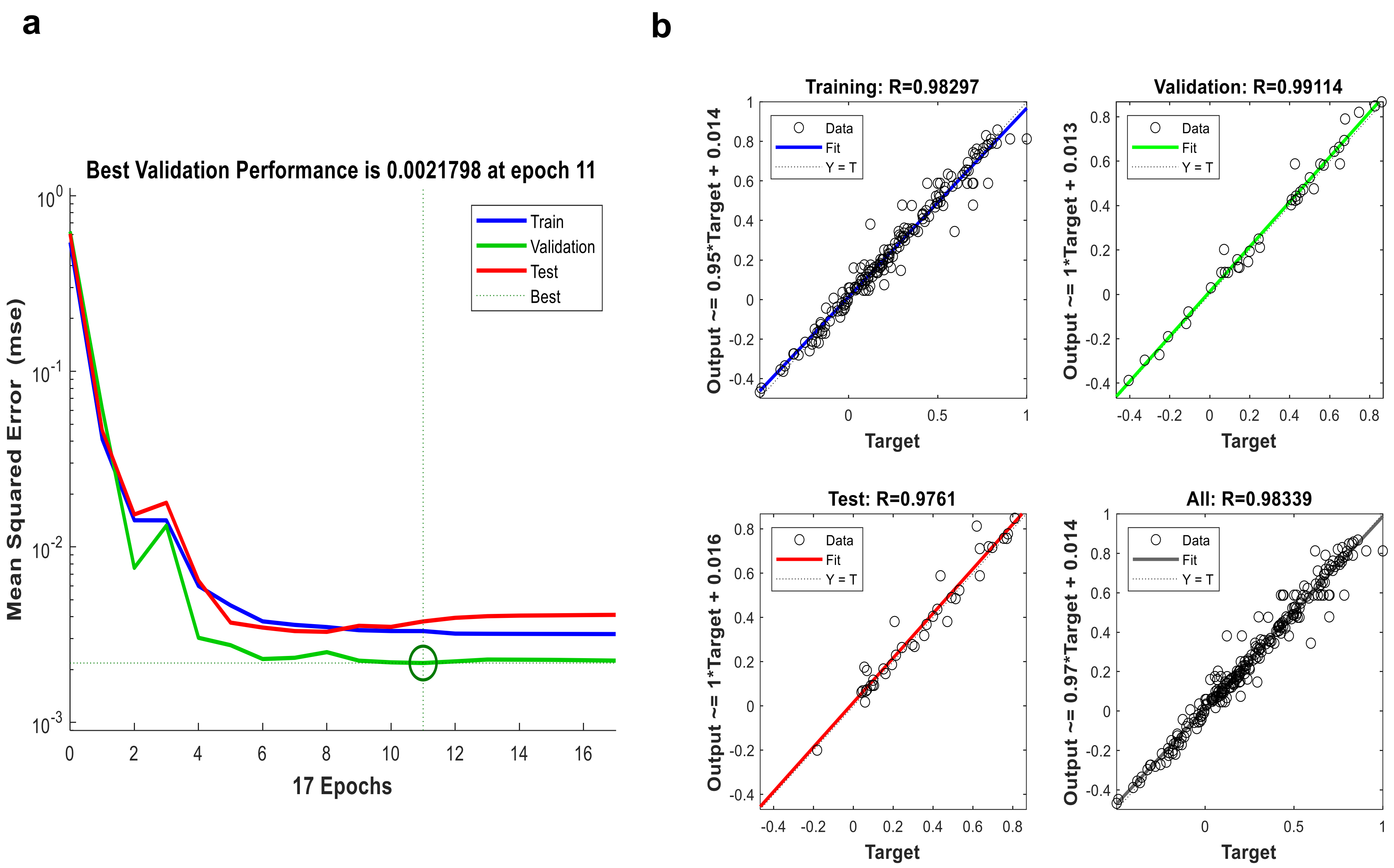

Supplementary Fig. 19: (a) Performance metric of ANN model showing the lowest MSE of 0.0021798 after 11 epochs. **(b)** and regression analysis of different data subset: test, training, validation and all of them combined

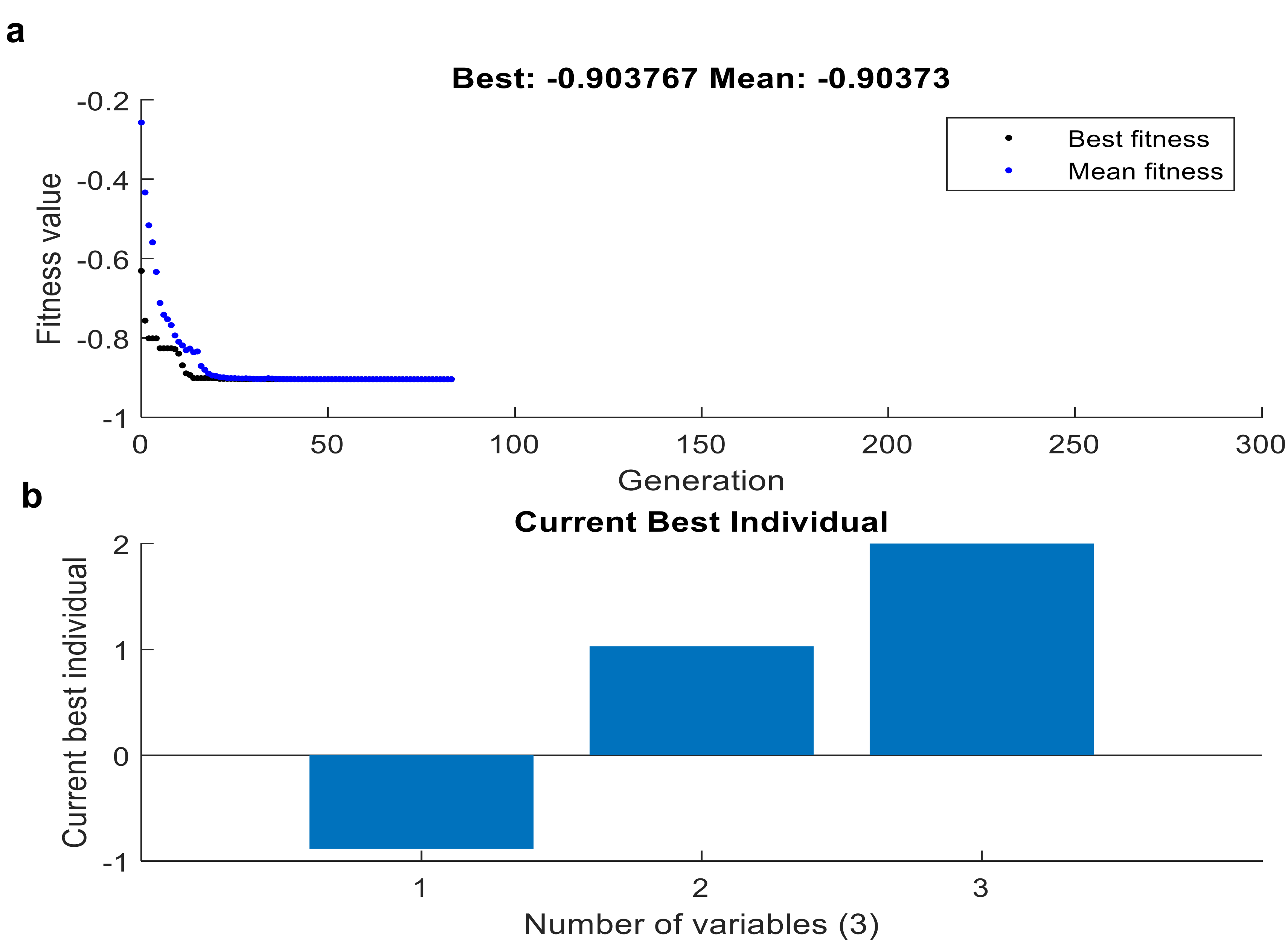

Supplementary Fig. 20: (a) Evolution of fitness values and **(b)** identification of optimal variables across 90 generations via Genetic Algorithm (GA)

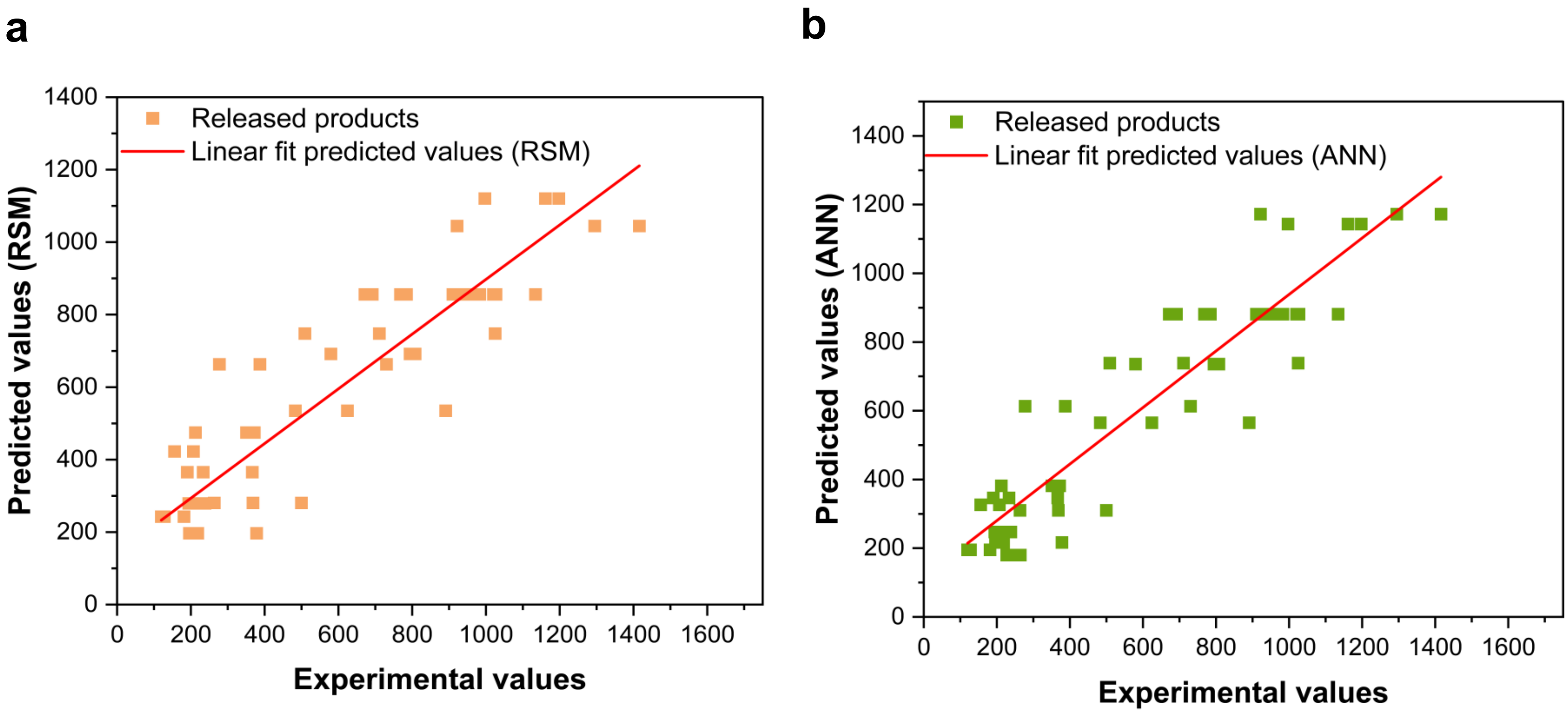

Supplementary Fig. 21: Comparative evaluation of predictive accuracy: Response Surface Methodology (RSM) vs. Artificial Neural Network (ANN) models (**a** and **b**, respectively), demonstrating improved prediction with the ANN model, which has a higher determination coefficient (0.84) compared to the RSM model (0.75).

References:

Ahmad MA, Alrozi R (2010) Optimization of preparation conditions for mangosteen peel-based activated carbons for the removal of Remazol Brilliant Blue R using response surface methodology. Chem Eng J 165:883–890. https://doi.org/10.1016/j.cej.2010.10.049

Alloun W, Berkani M, Benaissa A, Shavandi A, Gares M, Danesh C, Lakhdari D, Ghfar AA, Chaouche NK (2023) Waste valorization as low-cost media engineering for auxin production from the newly isolated *Streptomyces rubrogriseus* AW22: Model development. Chemosphere 326:138394. https://doi.org/10.1016/j.chemosphere.2023.138394

Guendouzi S, Benmati M, Bounabi H, Vicente Carbajosa J (2024) Application of response surface Methodology coupled with Artificial Neural network and genetic algorithm to model and optimize symbiotic interactions between *Chlorella vulgaris* and *Stutzerimonas stutzeri* strain J3BG for chlorophyll accumulation. Bioresour Technol 394:130148. https://doi.org/10.1016/j.biortech.2023.130148

Liu F, Wang T, Yang W, Zhang Y, Gong Y, Fan X, Wang G, Lu Z, Wang J (2023) Current advances in the structural biology and molecular engineering of PETase. Front Bioeng Biotechnol 11:1263996. https://doi.org/10.3389/fbioe.2023.1263996

Magalhães RP, Cunha JM, Sousa SF (2021) Perspectives on the Role of Enzymatic Biocatalysis for the Degradation of Plastic PET. Int J Mol Sci 22:11257. https://doi.org/10.3390/ijms222011257

Spiller B, Gershenson A, Arnold FH, Stevens RC (1999) A structural view of evolutionary divergence. Proc Natl Acad Sci 96:12305–12310. https://doi.org/10.1073/pnas.96.22.12305

Streit TM, Borazjani A, Lentz SE, Wierdl M, Potter PM, Gwaltney SR, Ross MK (2008) Evaluation of the “side door” in carboxylesterase-mediated catalysis and inhibition. Biol Chem 389:149–162. https://doi.org/10.1515/BC.2008.017
